# Large-Scale RNA Interference Screening in Mammalian Cells Identifies Novel Regulators of Mutant Huntingtin Aggregation

**DOI:** 10.1371/journal.pone.0093891

**Published:** 2014-04-04

**Authors:** Tomoyuki Yamanaka, Hon Kit Wong, Asako Tosaki, Peter O. Bauer, Koji Wada, Masaru Kurosawa, Tomomi Shimogori, Nobutaka Hattori, Nobuyuki Nukina

**Affiliations:** 1 Department of Neuroscience for Neurodegenerative Disorders, Juntendo University Graduate School of Medicine, Tokyo, Japan; 2 Laboratory for Structural Neuropathology, RIKEN Brain Science Institute, Saitama, Japan; 3 Laboratory for Molecular Mechanisms of Thalamus Development, RIKEN Brain Science Institute, Saitama, Japan; 4 Center for Neurologic Diseases, Department of Neurology, Brigham and Women's Hospital and Harvard Medical School, Harvard Institutes of Medicine, Boston, Massachusetts, United States of America; 5 Department of Neurology, Juntendo University Graduate School of Medicine, Tokyo, Japan; 6 CREST (Core Research for Evolutionary Science and Technology), JST, Tokyo, Japan; National Center of Neurology and Psychiatry, Japan

## Abstract

In polyglutamine (polyQ) diseases including Huntington's disease (HD), mutant proteins containing expanded polyQ stretch form aggregates in neurons. Genetic or RNAi screenings in yeast, *C. elegans* or *Drosophila* have identified multiple genes modifying polyQ aggregation, a few of which are confirmed effective in mammals. However, the overall molecular mechanism underlying polyQ protein aggregation in mammalian cells still remains obscure. We here perform RNAi screening in mouse neuro2a cells to identify mammalian modifiers for aggregation of mutant huntingtin, a causative protein of HD. By systematic cell transfection and automated cell image analysis, we screen ∼12000 shRNA clones and identify 111 shRNAs that either suppress or enhance mutant huntingtin aggregation, without altering its gene expression. Classification of the shRNA-targets suggests that genes with various cellular functions such as gene transcription and protein phosphorylation are involved in modifying the aggregation. Subsequent analysis suggests that, in addition to the aggregation-modifiers sensitive to proteasome inhibition, some of them, such as a transcription factor Tcf20, and kinases Csnk1d and Pik3c2a, are insensitive to it. As for Tcf20, which contains polyQ stretches at N-terminus, its binding to mutant huntingtin aggregates is observed in neuro2a cells and in HD model mouse neurons. Notably, except Pik3c2a, the rest of the modifiers identified here are novel. Thus, our first large-scale RNAi screening in mammalian system identifies previously undescribed genetic players that regulate mutant huntingtin aggregation by several, possibly mammalian-specific mechanisms.

## Introduction

Polyglutamine (polyQ) diseases are adult-onset hereditary neurodegenerative disorders. These include Huntington's disease (HD), spinocerebellar ataxias (SCA1, 2, 3, 6, 7, 17), dentatorubral-pallidoluysian atrophy (DRPLA) and spinobulbar muscular atrophy (SBMA). The polyQ diseases are caused by expansion of CAG repeats in certain causative genes. The mutant proteins containing expanded polyQ stretch are misfolded and aggregated, leading to formation of nuclear inclusions in neurons [1], [2].

The polyQ protein aggregation accompanies sequestration of several cellular components such as transcription factors [3]–[7] and RNA binding proteins [8], [9], leading to dysregulation of gene expression during neurodegeneration [10]–[12]. In addition, polyQ-mediated cell toxicity is reported to be reduced through suppressing polyQ aggregation by chaperones [13]–[18], chaperonin [19]–[21], QBP1 (polyQ-binding peptide 1) [22], [23], or chemical compounds such as Congo Red [24] or trehalose [25]. Thus, examination of molecular mechanisms underlying polyQ aggregation is one of the effective strategies for understanding pathomechanism of and searching therapeutic targets for polyQ diseases.

In past 10 years, several groups have performed genetic or RNA interference (RNAi) screening to identify polyQ aggregation-modifying genes using yeast [26], *C. elegans* [27]–[30] or *Drosophila* models [31]–[34]. These screenings have identified genes in various contexts such as transcription, RNA processing, protein transport and signal transduction, in addition to protein folding and degradation. These observations suggest that multiple cellular pathways are involved in the regulation of polyQ protein aggregation in non-mammalian systems. Although a few of their orthologues are shown to modify polyQ protein aggregation in mammalian cells [26], [34], a large-scale, systematic screen has not been performed in any mammalian systems and the overall molecular mechanism underlying polyQ protein aggregation in mammalian cells remains obscure.

To this end, we perform RNAi screening in mouse neuroblastoma cells to attempt to identify novel aggregation-modifiers for mutant huntingtin (Htt), a causative protein of HD, in mammals. To the best of our knowledge, this is the first comprehensive analysis of polyQ aggregation-modifying genes in mammals. We transduce ∼12000 short hairpin RNA (shRNA) clones into neuro2a cells that inducibly express mutant Htt, and analyze the aggregation by automated quantitative fluorescence microscopy. After three screenings, we identify 111 shRNAs that specifically modify the mutant Htt aggregation in neuro2a cells. Subsequent analyses suggest that the modifications can be mediated by several mechanisms, that is, by direct/indirect regulation through proteasome-dependent/-independent pathways. Importantly, all of the shRNA targets except of one gene [33] are not found by previous screenings using other organisms described above. Thus, our RNAi screening identifies previously undescribed genes involved in mutant Htt aggregation in mammalian cells.

## Results

### Identification of shRNAs that modify mutant Nhtt aggregation in neuro2a cells

**Figure 1:**
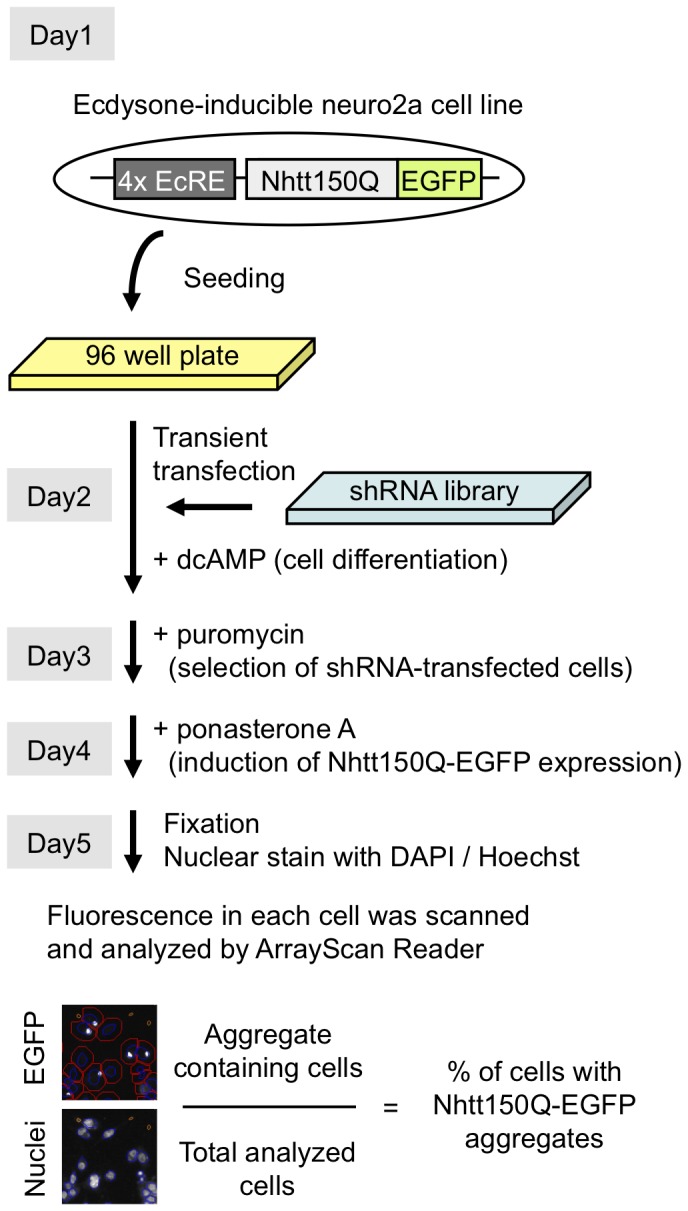
Experimental procedure of screening of shRNAs that modify Nhtt150Q-EGFP aggregation in neuro2a cells. Neuro2a cells inducibly expressing Nhtt150Q-EGFP under ecdysone-responsive element (EcRE) were seeded on a 96 well culture plate. On the next day, the cells were transiently transfected with shRNAs prepared from shRNA library plates. The cells were then differentiated by dcAMP on the same day and selected with puromycin on the next day, after which they were treated with ponasterone A to induce Nhtt150Q-EGFP expression. After 24 hr, the cells were fixed with 4% PFA and incubated with DAPI or Hoechst for nuclear staining. Fluorescence cell images were automatically obtained and analyzed by ArrayScan HCS Reader, and percent of cells with Nhtt150Q-EGFP aggregates among total analyzed cells was calculated.

To identify modifiers of mutant Htt aggregation in mammalian cells, we performed shRNA screening using mouse neuro2a cells that inducibly expressed exon 1 of Htt (Nhtt) containing a 150Q tagged with an EGFP at its C-terminus (Nhtt150Q-EGFP), under the control of ponasterone A [35]. shRNA libraries were purchased from Open Biosystems, in which shRNA clones were supplied as *E. coli* glycerol stocks in 96 well plate-formats. We used total 122 plates for plasmid DNA purification and obtained 11346 shRNA clones with transfection grade DNA. Scheme of experimental procedure is outlined in Figure 1. First, neuro2a cells were seeded on 96 well plates and transiently transfected with shRNA clones. Cells were then differentiated by dibutyryl cyclic AMP (dcAMP) on the same day and selected with puromycin on the next day. After the selection, they were treated with ponasterone A to induce Nhtt150Q-EGFP expression. EGFP positive aggregates will be allowed to form for one day. After fixation and nuclear staining with DAPI or Hoechst, the number of aggregates-containing cells and total number of cells were automatically quantified by Cellomics ArrayScan HCS Reader, a cell image analyzer equipped with fluorescence microscopy.

**Figure 2:**
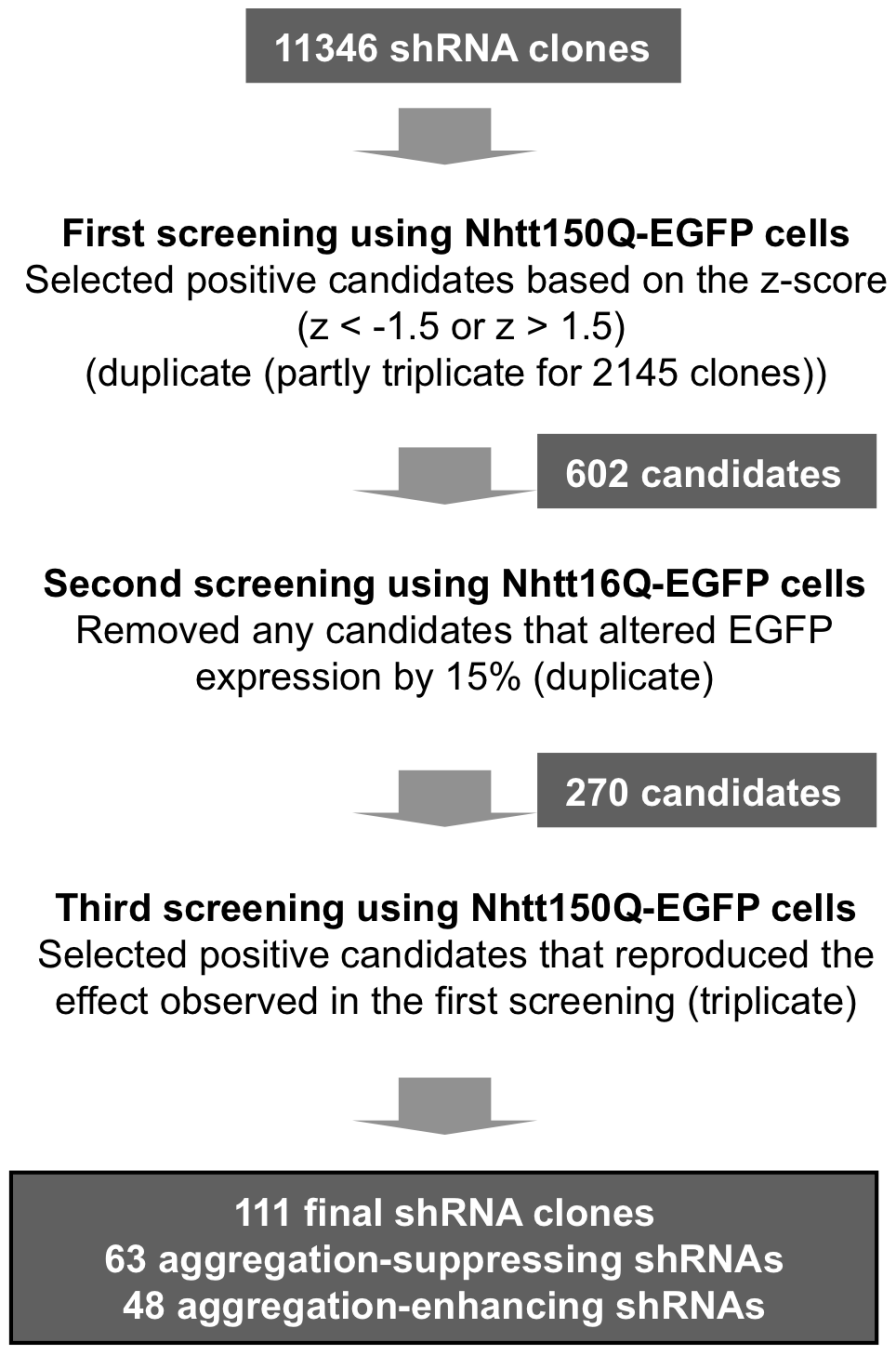
Summary of the shRNA screening. Total 11346 shRNA clones were subjected to the first screening using Nhtt150Q-EGFP cells. The screening was performed in duplicate (partly in triplicate), and shRNAs altering Nhtt150Q-EGFP aggregation at <−1.5 or >1.5 of z-score were selected as positive candidates. Obtained 602 clones were then subjected to the second screening using Nhtt16Q-EGFP cells to exclude the shRNAs modifying the aggregation by altering Nhtt expression itself. shRNAs altering EGFP intensity by 15% compared with the control were removed. Remaining 270 candidates were subjected to the third screening using Nhtt150Q-EGFP cells for confirmation (triplicate). We performed statistical analysis (t-test) in the third screening and shRNAs with *P*<0.1 were included as candidates. Finally, 111 shRNAs were obtained, among which 63 shRNAs suppressed and 48 shRNAs enhanced the Nhtt150Q-EGFP aggregation.

**Figure 3:**
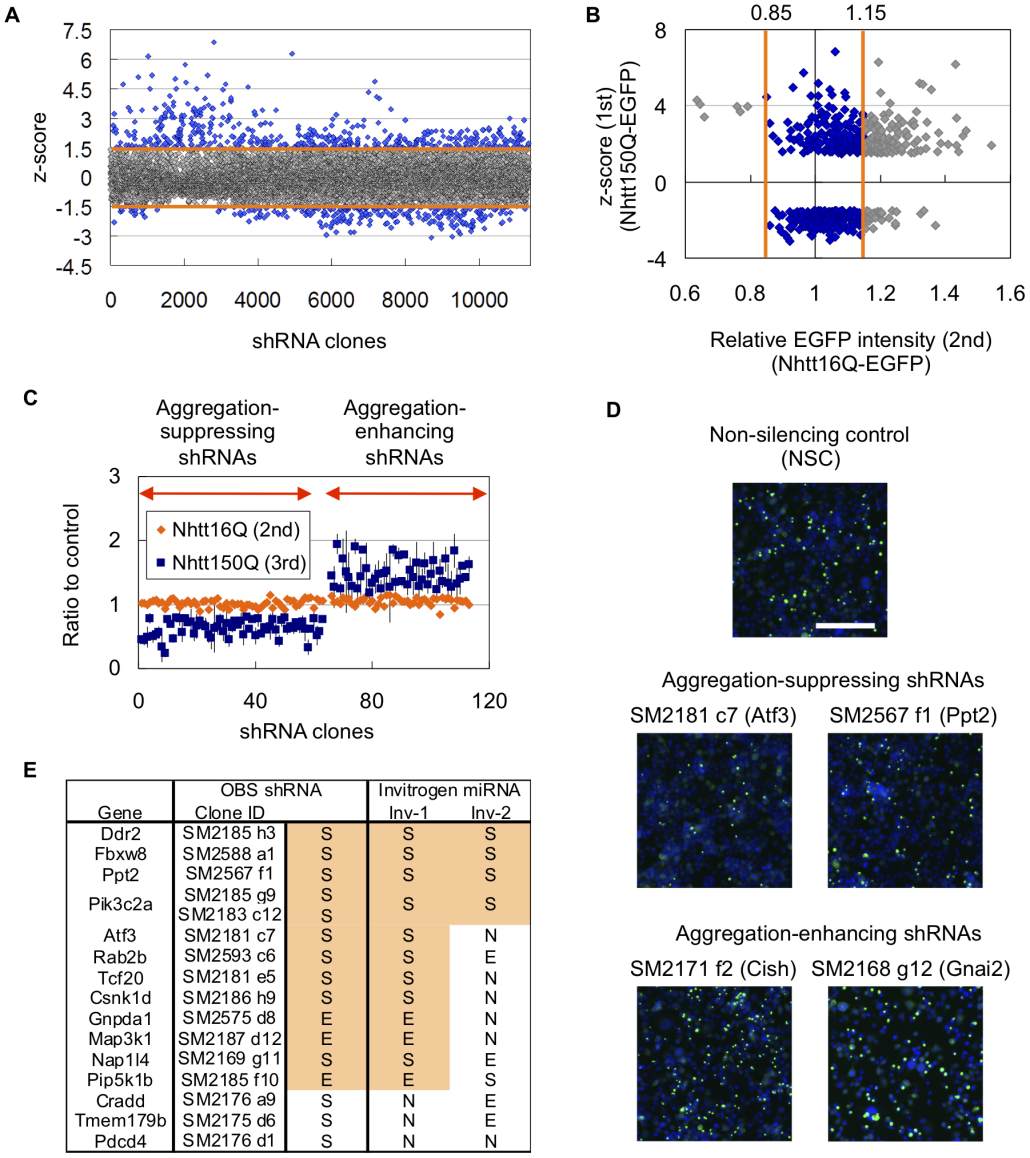
Identification and validation of the shRNAs modifying Nhtt150Q-EGFP aggregation in neuro2a cells. (A) Summarized data of the first screening using Nhtt150Q-EGFP cells. Mean z-score of 11346 shRNA clones were plotted. shRNAs showing the z-score outside the range of ±1.5 were picked up as positive candidates (indicated as blue plots). (B) Summarized data of the second screening using Nhtt16Q-EGFP cells. The data (relative EGFP intensity; x-axis) were plotted against the first screening data (z-score; y-axis) for 602 shRNA clones. The shRNAs showing the EGFP intensity within 15% to the control were picked up as positive candidates (indicated as blue plots). (C) Data of the second and third screening for final 111 shRNA candidates. 63 shRNAs suppressed and 48 shRNAs enhanced the Nhtt150Q-EGFP aggregation without distinct alteration of Nhtt16Q-EGFP expression. (D) Example cell images. Two shRNAs targeting Atf3 or Ppt2 reduced the cells with Nhtt150Q-EGFP aggregates compared with non-silencing control whereas two shRNAs targeting Cish or Gnaj2 increased them. Bar is 0.2 mm. (E) Validation of the effect of Open Biosystems (OBS) shRNA by two miRNAs (Inv-1 and -2) binding to different sequences of the genes (Data S2). The S, N and E mean suppression, no-effect and enhancement for the Nhtt150Q-EGFP aggregation, respectively. In case of Pik3c2a, two different shRNAs were obtained. Note that the shRNA's effect was reproduced by at least one miRNA in 12 genes (80% of analyzed genes).

The screening strategy is summarized in Figure 2. In the first screening, we screened 11346 shRNA clones based on the z score (mean z score is <−1.5 or >1.5) and finally obtained 602 shRNAs that were able to modify Nht150Q-EGFP aggregation in neuro2a cells (Figure 3A). To exclude the shRNAs that modify the aggregation purely acting on Nhtt expression itself, we performed a second screen, in which we used neuro2a cells expressing non-aggregating Nhtt16Q-EGFP [35]. After transfection and induction of Nhtt16Q-EGFP expression as above, EGFP intensities in the cells were quantified by ArrayScan reader. Through this analysis, we noticed that the shRNAs that induced Nhtt16Q-EGFP expression were relatively enriched in the aggregation-enhancing shRNAs (Figure 3B), suggesting that their enhancing effect was just caused by inducing Nhtt expression. After the second screen, 270 shRNAs were remained as candidates as they did not show significant alteration in Nhtt16Q-EGFP expression (Figure 3B). To obtain shRNAs that reproducibly modifying aggregation, we again used Nhtt150Q-EGFP cells for the third screening. Finally, we obtained 111 shRNAs that specifically and reproducibly modified Nhtt150Q-EGFP aggregation in neuro2a cells (Figure 3C).

### Validation, classification and proteasome-dependency of the aggregation-modification by the identified shRNAs

Among the final 111 shRNAs, 63 suppressed and 48 enhanced the aggregation of mutant Nhtt (Table 1, 2) (complete screening data for 111 candidates are described in Data S1). Figure 3D shows representative cell images of the aggregation-modification by the shRNAs; Atf3 or Ppt2 shRNAs reduced Nhtt-150QEGFP aggregates compared with non-silencing control whereas Cish or Gnai2 shRNAs increased them. To validate the gene-knockdown effect by the shRNAs, we designed other RNAi sequences (Inv-1 and -2) by Invitrogen's BLOCK-iT RNAi Designer for some of the genes (Data S2), and expressed as miRNA (miR RNAi) using pcDNA6.2-mRFP-miR vector [36]. We reproduced the aggregation-modifying ability in 12 out of 15 genes (80%) by at least one of the miRNAs (Figure 3E), supporting the validity of our screening strategy and results.

**Table 1:** shRNAs suppressing Nhtt150Q aggregation in neuro2a cells.

	shRNA		1st (150Q)	2nd (16Q)	3rd (150Q)	
No	Clone ID	Gene	z-score	Ratio	Ratio (+ SD)	Gene Description
1	SM2173 d5	1810032O08Rik	−1.79	1.03	0.447 (+0.084)	RIKEN cDNA 1810032O08 gene
2	SM2177 c4	4933425L06Rik	−2.17	1.02	0.447 (+0.160)	RIKEN cDNA 4933425L06 gene
3	SM2179 b7	Abat	−1.65	0.99	0.493 (+0.086)	4-aminobutyrate aminotransferase
4	SM2574 c8	Aktip	−1.60	1.03	0.787 (+0.090)	thymoma viral proto-oncogene 1 interacting protein
5	SM2181 c7	Atf3	−1.51	1.06	0.499 (+0.093)	activating transcription factor 3
6	SM2172 b5	Birc6	−2.29	1.07	0.533 (+0.098)	baculoviral IAP repeat-containing 6
7	SM2173 f4	Casp3	−1.52	1.02	0.563 (+0.237)	caspase 3
8	SM2174 e3	Cd24a	−2.45	1.03	0.339 (+0.066)	CD24a antigen
9	SM2176 a9	Cradd	−2.27	0.93	0.241 (+0.028)	death domain-containing protein, RAIDD
10	SM2186 h9	Csnk1d	−2.22	1.03	0.771 (+0.060)	casein kinase 1, delta
11	SM2185 h3	Ddr2	−1.52	1.09	0.699 (+0.069)	discoidin domain receptor family, member 2
12	SM2168 c8	Ddx6	−1.75	1.10	0.467 (+0.038)	DEAD (Asp-Glu-Ala-Asp) box polypeptide 6
13	SM2579 f9	Efhb	−2.60	1.05	0.793 (+0.122)	EF hand domain family, member B
14	SM2558 e11	Enah	−1.55	0.96	0.787 (+0.148)	enabled homolog (Drosophila)
15	SM2588 a1	Fbxw8	−1.57	1.03	0.564 (+0.056)	F-box and WD-40 domain protein 8
16	SM2561 e3	Fcgr3	−1.90	1.02	0.781 (+0.024)	Fc receptor, IgG, low affinity III
17	SM2176 h8	Fgd1	−1.92	1.06	0.719 (+0.200)	FYVE, RhoGEF and PH domain containing 1
18	SM2560 h6	Fkbp9	−2.34	1.01	0.736 (+0.117)	FK506 binding protein 9
19	SM2179 b5	Fsd1l	−1.62	1.04	0.548 (+0.199)	FSD1-like
20	SM2179 e8	Gaa	−2.25	0.99	0.664 (+0.095)	glucosidase, alpha, acid
21	SM2560 e6	Gjb2	−1.77	0.89	0.743 (+0.036)	gap junction protein, beta 2
22	SM2177 e1	Gpr37	−2.79	1.05	0.680 (+0.141)	G protein-coupled receptor 37
23	SM2588 f3	Grhpr	−2.14	1.07	0.654 (+0.129)	glyoxylate reductase/hydroxypyruvate reductase
24	SM2174 g6	Hoxd3	−1.96	0.92	0.527 (+0.167)	homeobox D3
25	SM2175 c6	Jagn1	−1.68	1.06	0.473 (+0.381)	jagunal homolog 1 (Drosophila)
26	SM2591 e6	Klhl7	−1.66	1.06	0.635 (+0.120)	kelch-like 7
27	SM2134 h10	LOC195242	−1.92	1.10	0.650 (+0.099)	
28	SM2112 g12	LOC195373	−1.59	0.98	0.763 (+0.150)	
29	SM2185 a7	Lrguk	−1.79	0.95	0.577 (+0.211)	leucine-rich repeats and GUK containing
30	SM2587 b3	Mab21l3	−1.67	1.03	0.702 (+0.115)	mab-21-like 3 (C. elegans)
31	SM2169 g11	Nap1l4	−1.57	0.92	0.455 (+0.072)	nucleosome assembly protein 1-like 4
32	SM2146 b6	Nlrp10	−1.54	1.02	0.641 (+0.058)	NLR family, pyrin domain containing 10
33	SM2575 b10	Notch4	−2.02	1.00	0.745 (+0.048)	notch 4
34	SM2176 f1	Olfr1339	−1.99	1.01	0.676 (+0.118)	olfactory receptor 1339
35	SM2008 a5	Olfr1451	−1.94	1.01	0.746 (+0.160)	olfactory receptor 1451
36	SM2174 c1	Olfr339	−2.96	0.92	0.526 (+0.062)	olfactory receptor 339
37	SM2142 b5	Olfr530	−1.89	0.99	0.808 (+0.042)	olfactory receptor 530
38	SM2588 f4	Olfr668	−2.65	1.00	0.623 (+0.049)	olfactory receptor 668
39	SM2562 d2	P2ry1	−1.94	0.97	0.764 (+0.136)	purinergic receptor P2Y, G-protein coupled 1
40	SM2176 d1	Pdcd4	−2.32	0.97	0.512 (+0.123)	programmed cell death 4
41	SM2575 a6	Pf4	−2.46	0.93	0.780 (+0.041)	platelet factor 4
42	SM2183 c12	Pik3c2a	−2.08	0.96	0.548 (+0.057)	PI 3-kinase, C2 domain containing, alpha
43	SM2185 g9	Pik3c2a	−1.99	1.01	0.637 (+0.043)	PI 3-kinase, C2 domain containing, alpha
44	SM2134 a11	Pkd1l3	−1.53	1.03	0.606 (+0.198)	polycystic kidney disease 1 like 3
45	SM2177 e5	Plec	−1.82	1.15	0.657 (+0.134)	plectin
46	SM2148 a11	Ppfia1	−1.58	0.95	0.775 (+0.107)	PTPRF, interacting protein (liprin), alpha 1
47	SM2567 f1	Ppt2	−2.15	1.06	0.605 (+0.093)	palmitoyl-protein thioesterase 2
48	SM2149 a9	Ptprn2	−2.03	1.02	0.434 (+0.103)	protein tyrosine phosphatase, receptor type, N2
49	SM2593 c6	Rab2b	−2.36	0.97	0.754 (+0.076)	RAB2B, member RAS oncogene family
50	SM2567 a8	Racgap1	−2.34	0.93	0.789 (+0.079)	Rac GTPase-activating protein 1
51	SM2008 h8	Rfc1	−1.65	1.09	0.614 (+0.074)	replication factor C (activator 1) 1
52	SM2588 d5	Rps27l	−1.58	1.06	0.660 (+0.087)	ribosomal protein S27-like
53	SM2169 b7	Slc25a14	−1.96	1.07	0.630 (+0.039)	solute carrier family 25member 14
54	SM2558 f9	Stfa3	−1.99	0.97	0.717 (+0.104)	stefin A3
55	SM2591 c7	Taf7l	−1.64	0.99	0.659 (+0.101)	TAF7-like RNA polymerase II, TBP-associated factor
56	SM2573 d3	Tbx18	−2.25	1.07	0.733 (+0.184)	T-box18
57	SM2181 e5	Tcf20	−1.94	1.11	0.613 (+0.114)	transcription factor 20
58	SM2175 d6	Tmem179b	−2.57	1.10	0.334 (+0.055)	transmembrane protein 179B
59	SM2591 c1	Tmem25	−1.61	1.11	0.620 (+0.099)	transmembrane protein 25
60	SM2141 e9	Trappc9	−1.62	0.95	0.778 (+0.149)	trafficking protein particle complex 9
61	SM2587 g7	Tspan10	−2.22	1.09	0.514 (+0.035)	tetraspanin 10
62	SM2149 a4	Txlna	−1.78	1.06	0.579 (+0.021)	taxilin alpha
63	SM2579 c2	Wdr37	−1.92	1.06	0.764 (+0.122)	WD repeat domain 37

List of 63 shRNAs suppressing Nhtt150Q-EGFP aggregation without distinct alteration of Nhtt16Q-EGFP expression in neuro2a cells. Targets genes of shRNAs and data summary of 1st (z-score), 2nd (ratio to control) and 3rd screening (ratio to control ± SD) are described. In case of Pik3c2a, two different shRNAs were obtained. Clone IDs of shRNAs are originally named in this paper based on plate number and well position of the shRNA.

**Table 2:** shRNAs enhancing Nhtt150Q aggregation in neuro2a cells.

			1st (150Q)	2nd (16Q)	3rd (150Q)	
No	Clone ID	Gene	z-score	Ratio	Ratio (+ SD)	Gene Description
1	SM2592 c7	Aimp2	2.24	1.13	1.446 (+0.331)	ARS interacting multifunctional protein 2
2	SM2023 a1	Aldh3b1	2.98	1.04	1.270 (+0.158)	aldehyde dehydrogenase 3 family, member B1
3	SM2598 f6	Arhgap24	1.57	1.05	1.945 (+0.103)	Rho GTPase activating protein 24
4	SM2108 a4	C1qtnf9	5.15	1.01	1.246 (+0.311)	C1q and tumor necrosis factor related protein 9
5	SM2171 f2	Cish	2.20	1.11	1.717 (+0.647)	cytokine inducible SH2-containing protein
6	SM2563 h3	Clec4e	1.86	1.07	1.512 (+0.387)	C-type lectin domain family 4, member e
7	SM2566 f12	Cml1	1.71	1.10	1.427 (+0.170)	camello-like 1
8	SM2022 g1	Cmpk1	1.89	1.01	1.283 (+0.120)	cytidine monophosphate (UMP-CMP) kinase 1
9	SM2179 c5	Cnih2	1.63	0.98	1.905 (+0.244)	cornichon homolog 2 (Drosophila)
10	SM2570 c11	Gba	1.88	1.04	1.242 (+0.297)	glucosidase, beta, acid
11	SM2110 c2	Gm10336	2.09	1.07	1.439 (+0.055)	predicted gene 10336
12	SM2168 g12	Gnai2	2.18	1.07	1.855 (+0.408)	G protein alpha inhibiting 2
13	SM2575 d8	Gnpda1	1.74	1.14	1.559 (+0.104)	glucosamine-6-phosphate deaminase 1
14	SM2014 g8	Hdac5	3.51	1.02	1.182 (+0.162)	histone deacetylase 5
15	SM2598 a11	Krcc1	2.22	1.06	1.340 (+0.225)	lysine-rich coiled-coil 1
16	SM2021 a4	Lmln	4.98	0.93	1.398 (+0.218)	leishmanolysin-like (metallopeptidase M8 family)
17	SM2111 e11	LOC210191	2.06	1.01	1.476 (+0.136)	
18	SM2113 d11	LOC226712	2.83	1.11	1.250 (+0.188)	
19	SM2187 d12	Map3k1	1.67	0.98	1.491 (+0.280)	mitogen-activated protein kinase kinase kinase 1
20	SM2173 a12	Mark3	1.52	1.15	1.524 (+0.554)	MAP/microtubule affinity-regulating kinase 3
21	SM2577 h12	Mei1	1.97	1.15	1.278 (+0.093)	meiosis defective 1
22	SM2561 g8	Myl12b	3.45	1.03	1.850 (+0.131)	myosin, light chain 12B, regulatory
23	SM2576 e12	Myo19	1.57	1.13	1.345 (+0.124)	myosin XIX
24	SM2580 c2	Mypop	1.99	1.10	1.768 (+0.066)	Myb-related transcription factor, partner of profilin
25	SM2563 f11	P2rx7	1.60	1.05	1.357 (+0.036)	purinergic receptor P2X, ligand-gated ion channel, 7
26	SM2583 c6	Pcdhb18	1.58	1.05	1.776 (+0.165)	protocadherin beta 18
27	SM2593 d2	Pcgf3	2.47	1.06	1.307 (+0.198)	polycomb group ring finger 3
28	SM2561 h8	Pcp2	2.13	1.00	1.539 (+0.278)	Purkinje cell protein 2 (L7)
29	SM2185 f10	Pip5k1b	2.02	1.02	1.423 (+0.324)	phosphatidylinositol-4-phosphate 5-kinase, type 1 beta
30	SM2563 h5	Plp2	1.84	1.11	1.652 (+0.352)	proteolipid protein 2
31	SM2022 g10	Ptpn11	2.42	1.00	1.292 (+0.176)	protein tyrosine phosphatase, non-receptor type 11
32	SM2109 c7	Rassf4	3.49	1.08	1.382 (+0.167)	Ras association domain family member 4
33	SM2139 h11	Rnf20	2.06	1.09	1.644 (+0.143)	ring finger protein 20
34	SM2109 e10	Serpinb10	3.50	1.04	1.315 (+0.178)	serine peptidase inhibitor, clade B, member 10
35	SM2598 h10	Slain2	2.25	0.98	1.479 (+0.284)	SLAIN motif family, member 2
36	SM2583 c9	Snx10	1.83	1.10	1.691 (+0.168)	sorting nexin 10
37	SM2568 e11	Spa17	1.86	1.09	1.233 (+0.191)	sperm autoantigenic protein 17
38	SM2112 g7	Spata21	4.45	0.85	1.523 (+0.099)	spermatogenesis associated 21
39	SM2568 h10	St6galnac2	1.65	1.11	1.359 (+0.195)	ST6-N-acetylgalactosaminide alpha-2,6-sialyltransferase 2
40	SM2168 f8	Stxbp1	1.52	1.08	1.713 (+0.303)	syntaxin binding protein 1
41	SM2568 d8	Sult2a2	1.59	1.11	1.338 (+0.253)	sulfotransferase family 2A
42	SM2565 c8	Svep1	2.88	1.08	1.274 (+0.266)	sushi, EGF and pentraxin domain containing 1
43	SM2184 a10	Syk	2.91	0.95	1.844 (+0.251)	spleen tyrosine kinase
44	SM2559 f11	Syt10	1.74	1.07	1.322 (+0.248)	synaptotagmin X
45	SM2142 g10	Tbc1d10c	3.38	1.07	1.402 (+0.046)	TBC1 domain family, member 10c
46	SM2587 a10	Tmem63a	1.71	1.05	1.604 (+0.054)	transmembrane protein 63a
47	SM2565 b8	Trem1	1.95	1.06	1.419 (+0.125)	triggering receptor expressed on myeloid cells 1
48	SM2583 e7	Wdpcp	2.38	1.00	1.627 (+0.187)	WD repeat containing planar cell polarity effector

List of 48 shRNAs enhancing Nhtt150Q-EGFP aggregation without distinct alteration of Nhtt16Q-EGFP expression in neuro2a cells. Targets genes of shRNAs and data summary of 1st (z-score), 2nd (ratio to control) and 3rd screening (ratio to control ± SD) are described. Clone IDs of shRNAs are originally named in this paper based on plate number and well position of the shRNA.

**Figure 4:**
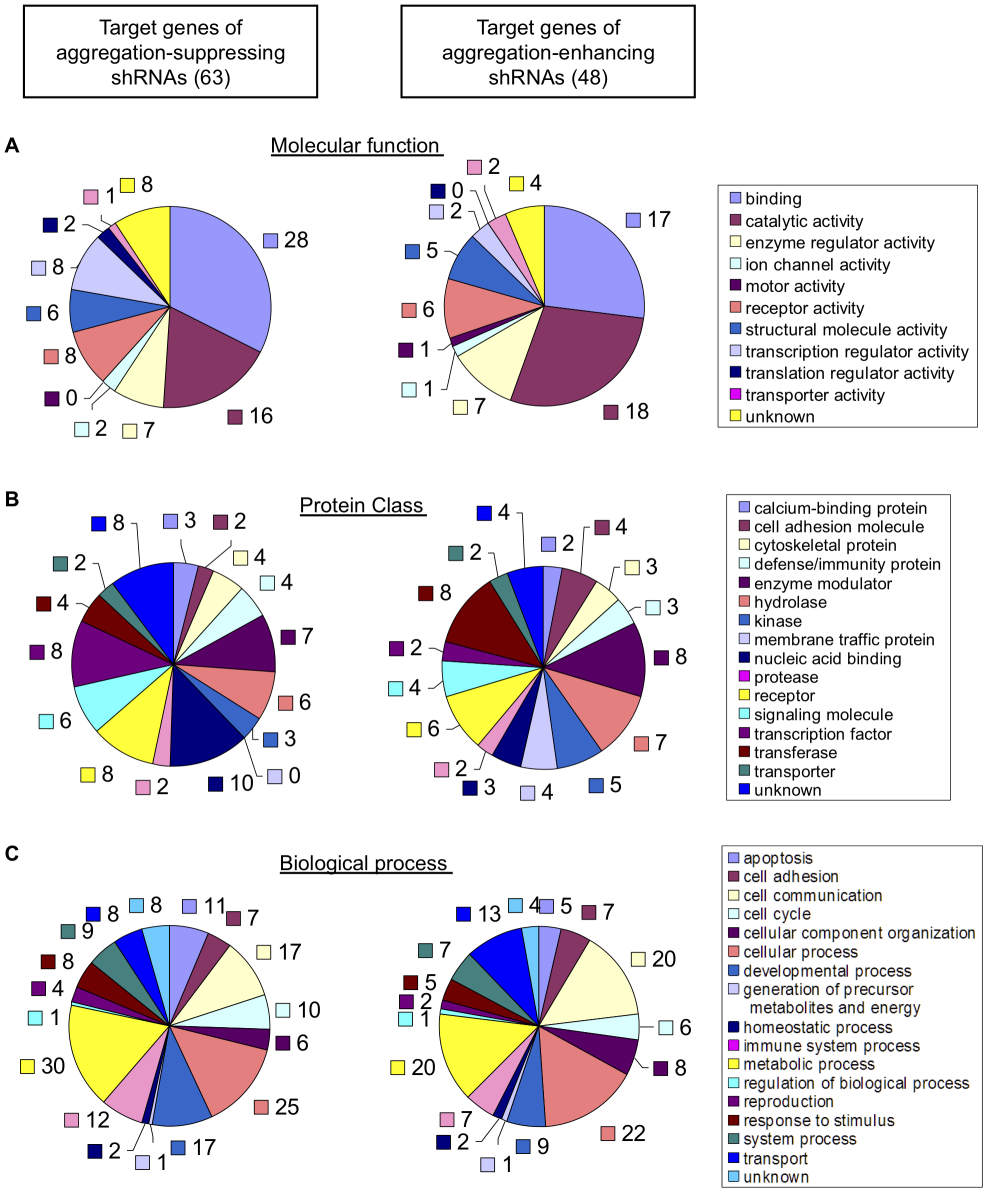
Classification of the shRNA-target genes. Target genes of the aggregation-suppressing (left chart) or -enhancing (right chart) shRNAs were classified using PANTHER Classification System based on molecular function (A), PANTHER Protein Class (B), or biological process (C). Genes not found in the database were classified as unknown. The numbers of classified genes were described.

To examine the molecular mechanism underlying mutant Nhtt aggregation by these shRNAs, we classified the final 111 shRNA-target genes using PANTHER Classification System [37]. When focused on molecular function, they were classified into various functions such as catalytic, receptor and transcription regulator activities (Figure 4A). Classification by PANTHER protein class also suggests the genes with various activities involved in mutant Nhtt aggregation (Figure 4B). Another Classification method further suggests the involvement of multiple biological processes in the aggregation modification (Figure 4C). Although no marked difference was observed between the target genes of the aggregation-suppressing and -enhancing shRNAs, transcription factors such as Atf3 and Tcf20 was more abundant in the suppressors' targets (12.7%; 8 out of 63) than the enhancers' targets (4.1%; 2 out of 48) (Figure 4A, B). In contrast, genes for membrane trafficking such as Stxbp1 and Snx10 were only found in the enhancers' targets (8.3%; 4 out of 48) (Figure 4B). We also performed Statistical Overrepresentation Test using PANTHER Classification System, however any of gene ontology (GO) term or pathway was not significantly enriched (data not shown). These data suggest that genes with broad molecular and biological functions modify the mutant Nhtt aggregation whereas some specific cellular functions such as gene transcription and membrane trafficking may be differentially involved in the modification.

**Figure 5:**
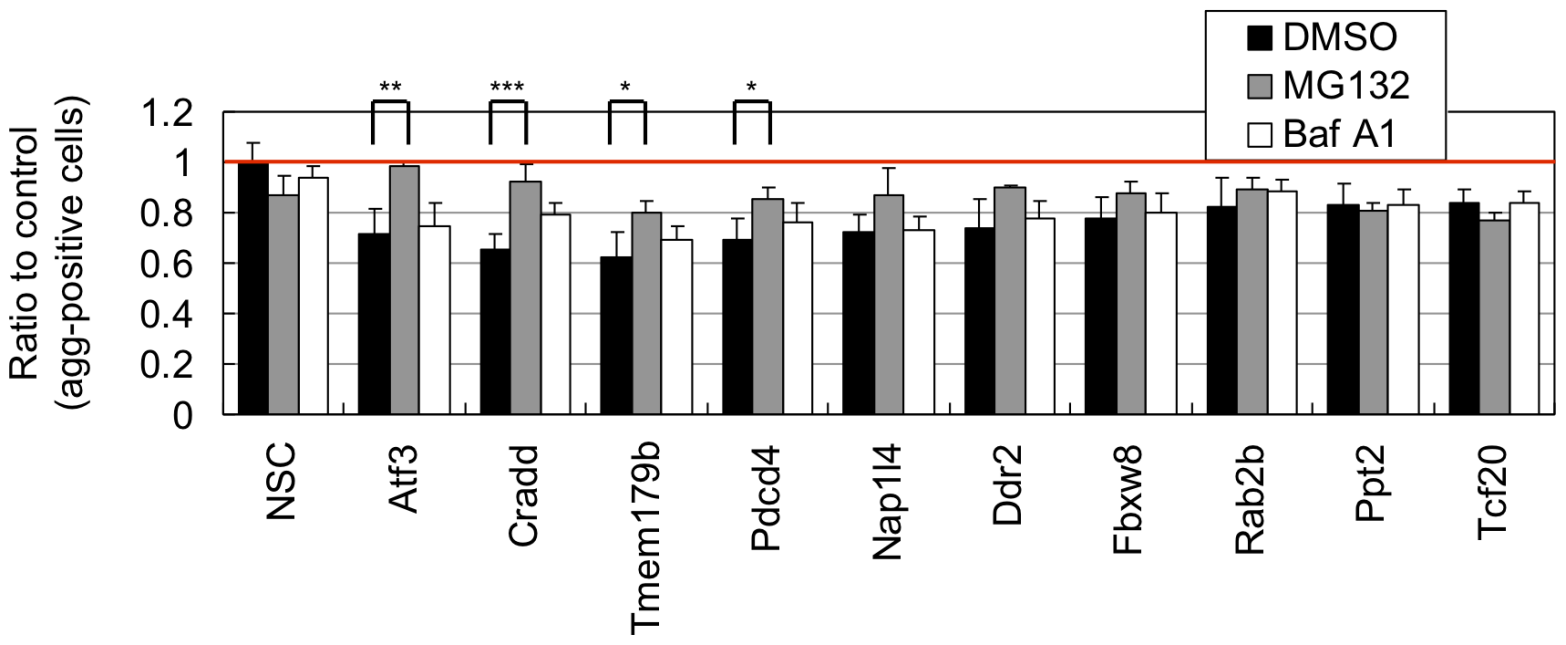
Effect of proteasome or autophagy inhibitor on shRNA-mediated modification of Nhtt150Q-EGFP aggregation. Nhtt150Q-EGFP cells transfected with control (NSC) or shRNAs for indicated genes were treated with 0.5 μM MG132, 0.5 μM Baf A1 or DMSO together with ponasterone A for 24 hr. The cells with Nhtt150Q-EGFP aggregates were quantified by ArrayScan reader. Some of shRNA's suppressive effects on the aggregation were relieved by treatment with MG132 but not with Baf A1. Values are means ± SD of four well data (*P<0.05, **P<0.01, ***P<0.001).

Several lines of studies have suggested an involvement of ubiquitin-proteasome system, a major protein degradation system in cells, in mutant Htt degradation [34], [38]–[41]. In addition, another degradation system, autophagy, is also recently shown to be involved in clearance mutant Htt aggregates [36], [42]–[45]. To examine whether the aggregation-modification by identified shRNAs involves these systems, we picked up 10 aggregation-suppressing shRNAs whose targets have various molecular and biological functions (Figure 4), and examined their effect in the presence of MG132 or bafilomycin A1 (Baf A1), inhibitor of proteasome or autophagy, respectively. We found that MG132 but not Baf A1 relieved the aggregation-suppressing effect of several shRNAs, such as those for Atf3, Cradd, Tmem179b and Pdcd4 (Figure 5), suggesting that these genes modify the mutant Nhtt aggregation through proteasome-dependent mechanism.

### Tcf20 binds to mutant Nhtt aggregates in neuro2a cells and R6/2 mouse brain neurons

**Figure 6:**
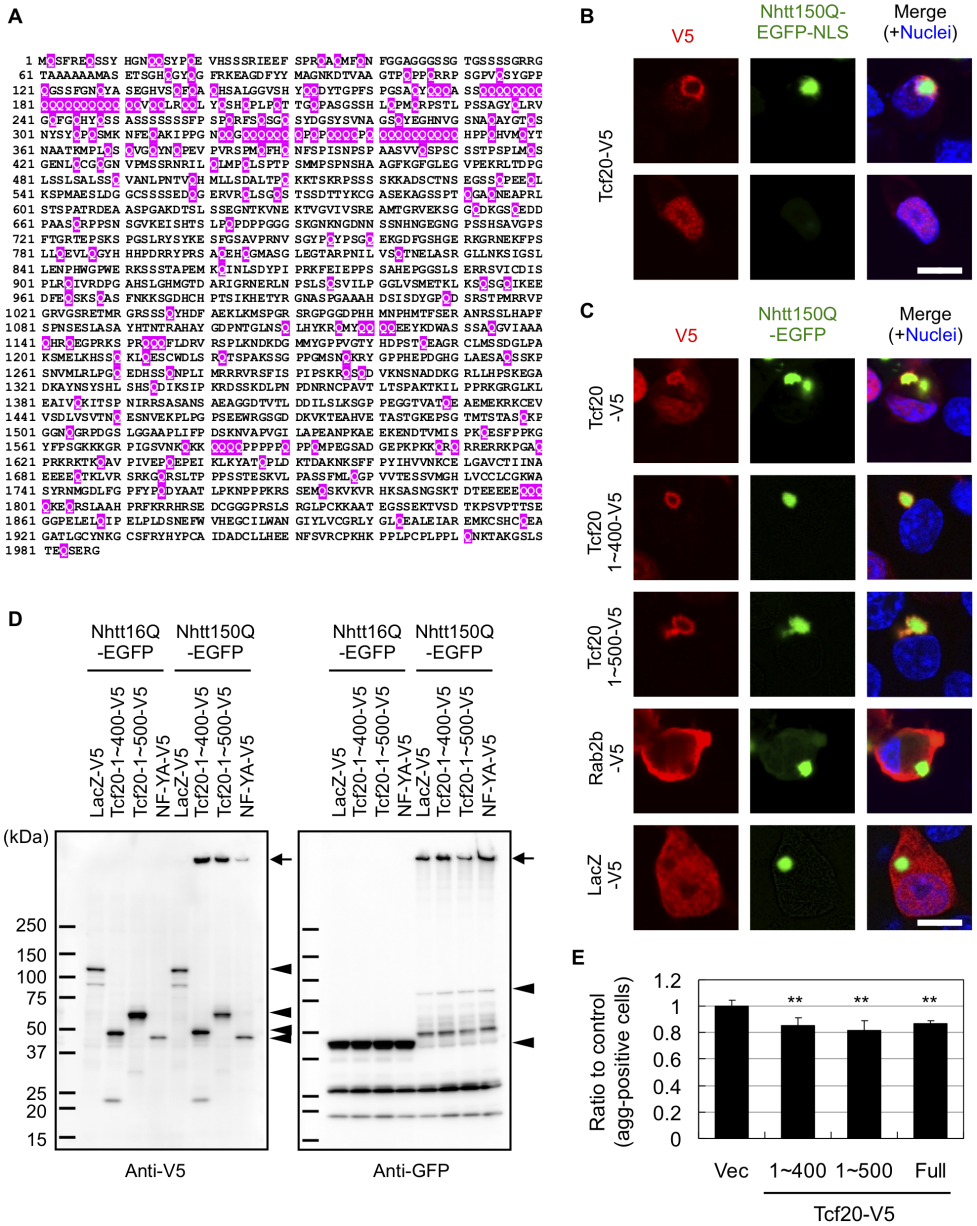
Tcf20 interacts with mutant Nhtt aggregates through its N-terminal region in neuro2a cells. (A) Primary sequences of mouse Tcf20. Glutamine (Q) residues were marked by red. Note that several polyQ stretches are observed in its N-terminal region. (B, C) Neuro2a cells were transfected with expression vectors for Nhtt150Q-EGFP-NLS (B) or Nhtt150Q-EGFP (C) together with those for indicated V5-tagged proteins. After one day, the cells were fixed and stained with anti-V5 antibody and Alexa 546-conjugated anti-mouse IgG. Nuclei were stained with DAPI. (B) Tcf20 co-accumulated with Nhtt150Q-EGFP-NLS aggregate in nucleus whereas it diffusely located in the nucleus without the aggregate. (C) Tcf20 or its N-terminal fragments accumulated with Nhtt150Q-EGFP aggregates in cytoplasm whereas Rab2b or LacZ did not. (D) Neuro2a cells were co-transfected with Nhtt150Q-EGFP expression vectors together with those for indicated V5-tagged proteins. After one day, the cells were subjected to Western blot analysis using anti-V5 and anti-GFP antibodies. Bands for expressed proteins are indicated by arrowheads. Note that gel top bands (indicated by arrows) were observed for Tcf20 (1∼400 or 1∼500)-V5 and NF-YA-V5 but not for LacZ-V5 by co-expression of Nhtt150Q-EGFP, suggesting their co-insolubilization with mutant Nhtt in neuro2a cells. (E) Nhtt150Q-EGFP cells were transfected with expression vectors for Tcf20 (full, 1∼400 or 1∼500)-V5 together with pRFP-C1 vector. After one day, cells were treated with ponasterone A for 24 hr and the cells with aggregates in trasnfected (RFP-positive) cells were quantified by ArrayScan reader. Ratios to control (empty vector) are shown. Values are means ± SD of four well data (**P<0.01). Scale bars are 10 μm (B, C).

In addition to the genes whose shRNAs' effect was sensitive to MG132 as described above, we found several genes including Ppt2 and Tcf20 whose shRNAs' effect was insensitive to it (Figure 5), suggesting proteasome-independent modification by these genes. Among them, we focused on Tcf20 because it is relatively Q-rich (Q composition is 9.7%) among the identified modifiers (mean Q composition is 4.59±1.92%) and notably it contains several polyQ stretches in the N-terminal region (Figure 6A). Because some of Q-rich proteins are shown to directly co-aggregate with mutant Htt [3]–[6], [8], [9], it is possible that Tcf20 directly interacts with mutant Nhtt aggregates.

**Figure 7:**
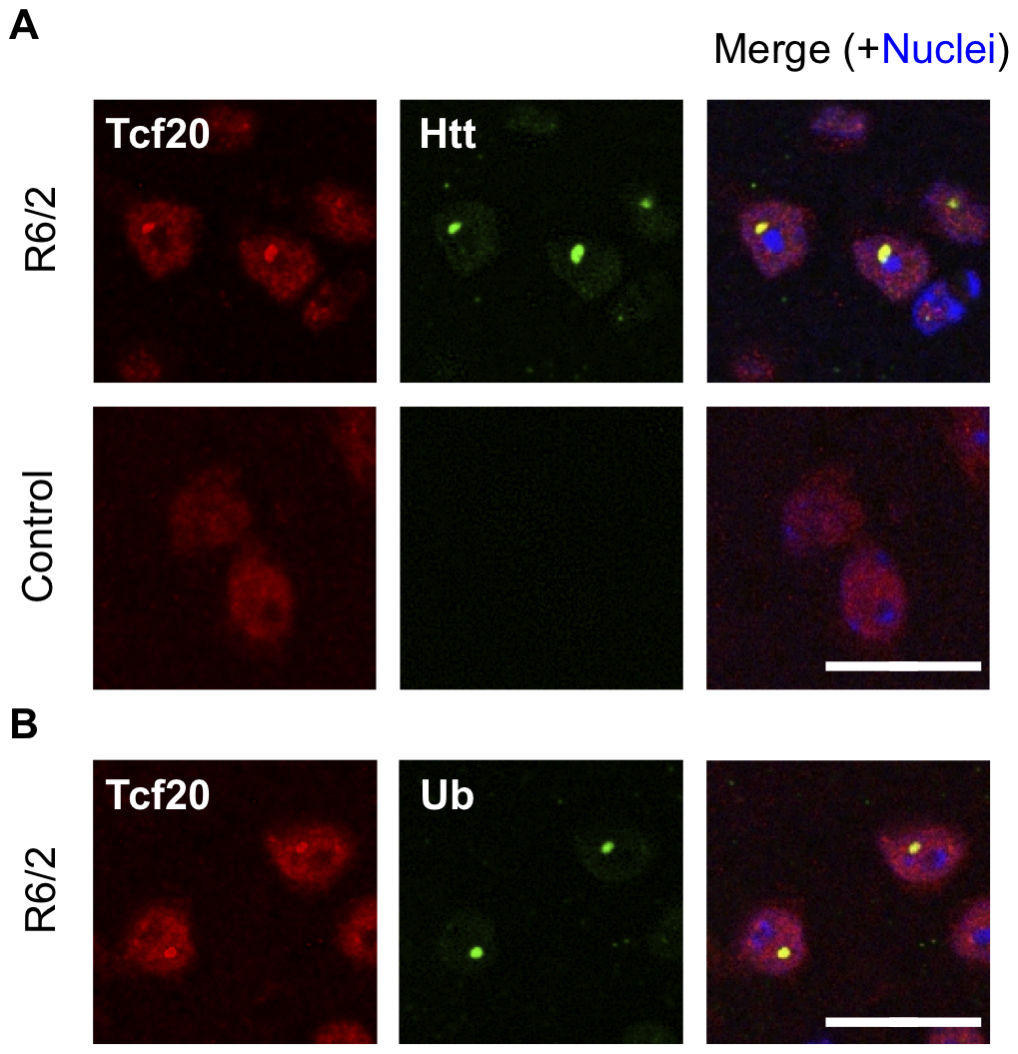
Anti-Tcf20 antibody stained nuclear inclusions in R6/2 mouse brain. Coronal brain sections (10 μm thick) of 12-week-old R6/2 or control male mouse were co-stained with anti-Tcf20 and anti-Htt (A) or anti-Ub (B). Nuclei were stained with DAPI. Cortical neurons are shown. Note that nuclear inclusions positive for Htt and Ub were stained by anti-Tcf20. Scale bars are 20 μm.

To examine this possibility, we cloned Tcf20 cDNA into pcDNA-DEST40 vector with a V5 tag at its C-terminus, and expressed Tcf20-V5 in neuro2a cells together with Nhtt150Q-EGFP-NLS (nuclear localization signal). As shown in Figure 6B, Tcf20-V5 was clearly co-localized with Nhtt150Q-EGFP-NLS aggregates, whereas it was only diffusely localized in the nucleus in the cells without the aggregates. Tcf20-V5 was also co-localized with cytoplasmic Nhtt150Q-EGFP aggregates (Figure 6C). In contrast, these co-localization was not observed for LacZ or other potential modifiers without polyQ-stretch such as Rab2b and Ddr2 (Q compositions are 5.6% and 4.0%, respectively) (Figure 6C; Ddr2, data not shown). We further found that Tcf20 N-terminal constructs (1∼400 and 1∼500) containing polyQ stretches preferentially co-localized with Nhtt150Q-EGFP aggregates (Figure 6C). In addition, these were insolubilized with Nhtt150Q-EGFP but not with Nhtt16Q-EGFP (Figure 6D), similar to a known aggregates-interacting protein, NF-YA [5]. Interestingly, overexpression of either N-terminal (1∼400 and 1∼500) or full-length of Tcf20 suppressed Nhtt150Q-EGFP aggregation (Figure 6E), suggesting that the Tcf20 interaction through its N-terminus suppresses mutant Nhtt aggregation when overexpressed. Finally, an antibody against Tcf20 stained puncta positive for Htt and Ub in cortical neurons of HD model mouse (Figure 7A, B), suggesting *in vivo* incorporation of Tcf20 into inclusions containing mutant Nhtt aggregates. These data suggest that the Tcf20 specifically interacts with mutant Nhtt aggregates through its N-terminus containing polyQ stretches to be incorporated into the inclusion. Because both knockdown and overexpression showed suppressive effect on mutant Nhtt aggregation, moderate expression of Tcf20 might be appropriate for efficient aggregation. Alternatively, its direct interaction and transcriptional activity could be differentially involved in the regulation of mutant Nhtt aggregation.

We have previously performed mass spectrometry of Nhtt150Q-EGFP aggregates purified from neuro2a cells and identified several aggregate-interacting proteins such as ubiquilin-1,-2, FUS/TLS and NF-YA/-YC [5], [8], [46]. By re-checking the data, we noticed that Tcf20 was contained in the mass spectrometry data. In addition, we found other modifiers, Hdac5 and Arhgap24, in the data, suggesting they are also the proteins incorporated into the aggregates. Analysis of their amino acid sequences revealed that Hdac5 but not Arhgap24 is relatively Q-rich (Q compositions are 8.9% and 4.8%, respectively). We also analyzed the Q composition of rest of the modifiers, however protein significantly in Q-rich like Tcf20 or Hdac5 was not found (data not shown). Taken together, these data suggest that Tcf20 and potentially Hdac5 are the proteins directly interacting with mutant Nhtt aggregates among the identified aggregation-modifiers.

### Suppression of mutant Nhtt aggregation by knocking down of Csnk1d and Pik3c2a

**Figure 8:**
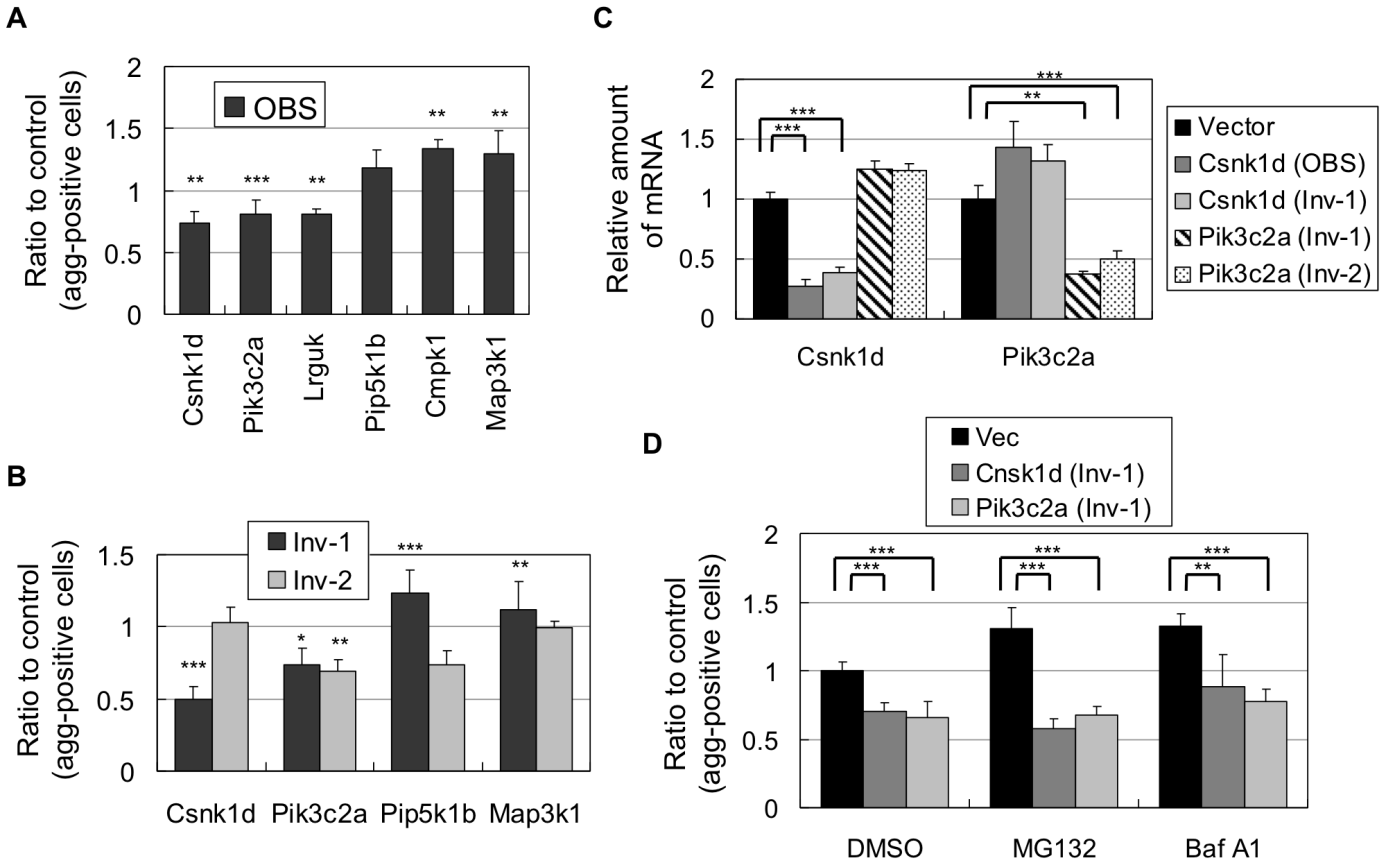
Kinases whose knockdown modifies Nhtt150Q-EGFP aggregation in neuro2a cells. (A, B) Nhtt150Q-EGFP cells were transfected with miRNA expression vector. After 2 days, cells were treated with ponasterone A for 24 hr and the cells with aggregates in miRNA trasnfected cells were quantified by ArrayScan reader. Ratios to control (empty vector) are shown. Note the reproduction of the shRNA's effect by miRNA binding to same sequence (OBS) (A) or difference sequences (Inv-1 or -2) (B). (C) Neuro2a cells expressing indicated miRNAs were subjected to quantitative RT-PCR. (D) Nhtt150Q-EGFP cells expressing indicated miRNAs were treated with 0.5 μM MG132, 0.2 μM Baf A1 or DMSO for 24 hr, and the cells with aggregates were quantified as above. Ratios to control (empty vector) are shown. Values are means ± SD of at least three well data (A–C) or six well data (D) (*P<0.05, **P<0.01, ***P<0.001).

To identify other molecular mechanisms regulating mutant Nhtt aggregation, we focused on kinases, key regulators of intracellular signal transduction. Our screening identified several kinases as potential modifiers for mutant Nhtt aggregation (Table 1, 2). These include Csnk1d, Pik3c2a and Lrguk, whose shRNAs suppressed the aggregation, and Cmpk1, Map3k1 and Pip5k1b, whose shRNAs enhanced it. The aggregation-modifying effect of these shRNAs can be reproduced when we used our own miRNAs that bind to same region as shRNA (OBS miRNA; Figure 8A), or different region (Inv-1 or -2 miRNA; Figure 1E, 8B) supporting the validity of gene knockdown effect of these kinases on the aggregation-modification. We then focused on two kinases, Csnk1d and Pik3c2a, whose knockdown suppressed Nhtt150Q-EGFP aggregation, and confirmed significant and specific reduction of gene expressions by their miRNAs (Figure 8C). We further examined the dependency of their knockdown effect on proteasome or autophagic activity, and found that their miRNAs were still effective even in the presence MG132 or Baf A1 (Figure 8D). Taken together, these data suggest that Csnk1d and Pik3c2a are involved in the modification of mutant Nhtt aggregation through proteasome- and autophagy-independent mechanisms.

### Knockdown of aggregation-modifiers did not suppress mutant Nhtt-induced cell toxicity

**Figure 9:**
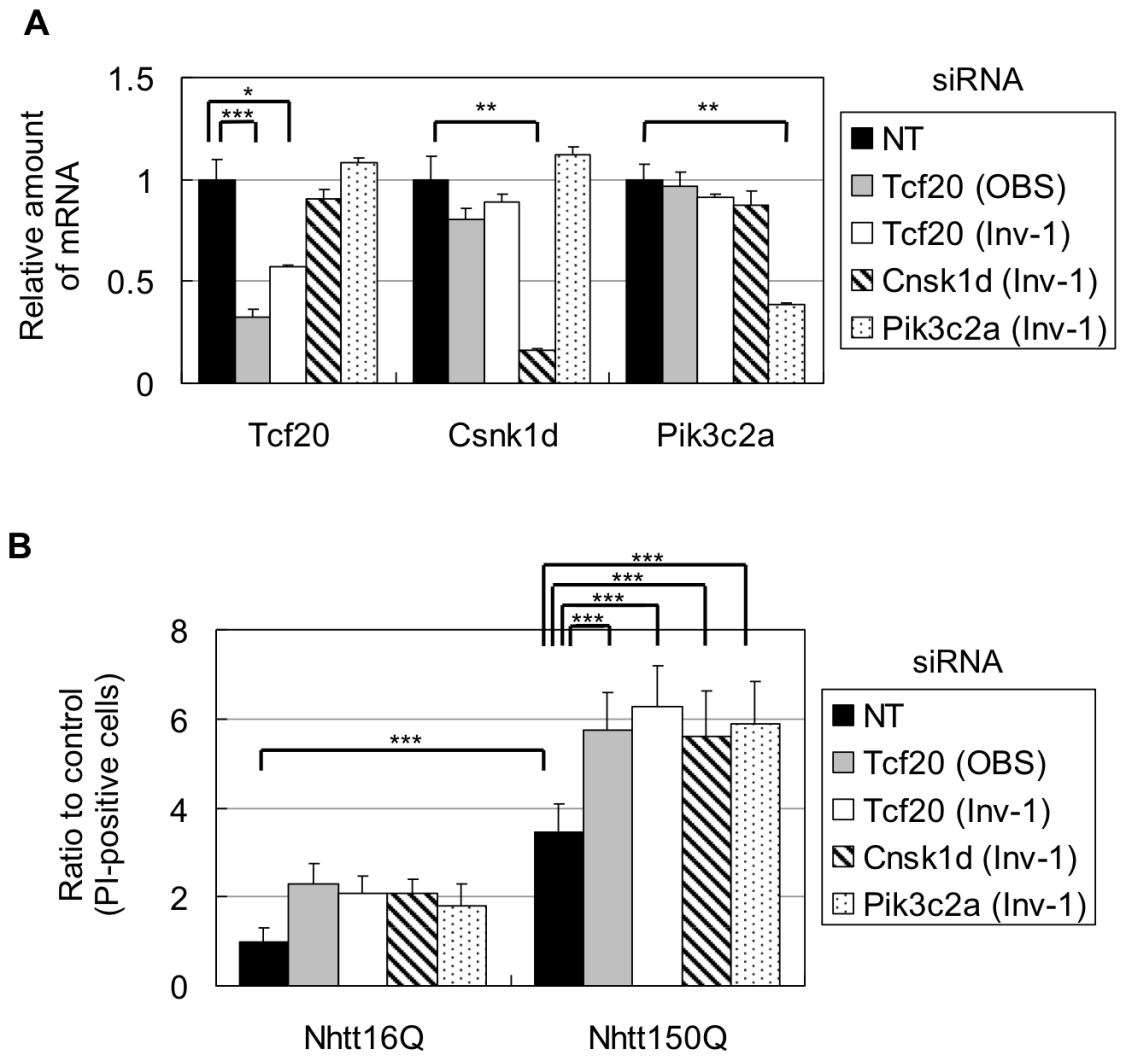
Effect of knockdown of aggregation-modifiers on mutant Nhtt-induced cell toxicity. (A) Neuro2a cells were transfected with siRNAs for NT (non-targeting), Tcf20 (OBS or Inv-1), Csnk1d (Inv-1) or Pik3c2a (Inv-1). After two days, the cells subjected to quantitative RT-PCR. Specific and significant reduction of their target mRNAs was observed. Values are means ± SD of three well data (*P<0.05, **P<0.01, *** *P*<0.001). (B) siRNA-transduced neuro2a cells were further transfected with expression vector for Nhtt16Q-EGFP or Nhtt150Q-EGFP. After two days, the cells were co-stained with PI and Hoechst and subjected to ArrayScan analysis to count PI-positive dead cells among the cells positive for both Hoechst and GFP. Ratios to control (NT siRNA- and Nhtt16Q-EGFP-transfected cells) were shown. Values are means ± SD of eight well data (***P<0.001).

Finally, we examined the effect of knockdown of Tcf20, Csnk1d or Pik3c2a on cell toxicity induced by mutant Nhtt. For this purpose, we first synthesized following siRNA oligos based on the miRNA sequences used above; two for Tcf20 (OBS and Inv-1) and one for Csnk1d (Inv-1) or Pik3c2a (Inv-1). We first confirmed that transduction of these siRNA induced significant and specific reduction of their target genes compared with that of non-targeting control (NT) in neuro2a cells (Figure 9A). We then transiently overexpressed Nhtt16Q-EGFP or Nhtt150Q-EGFP in siRNA-transduced cells. After two days, the cells were incubated with pyridinium iodide (PI) to detect dead cells, and percent of PI-positive cells per GFP-positive, Nhtt expressing cells was calculated by ArrayScan. As shown in Figure 9B, overexpression of Nhtt150Q-EGFP induced ∼3-fold increase in PI-positive cells compared with that of Nhtt16Q-EGFP, suggesting the induction of cell toxicity by mutant Nhtt in neuro2a cells. Notably, knockdown of Tcf20, Csnk1d or Pik3c2a did not suppress the toxicity rather enhanced it, although their knockdown seemed to be also effective, but to a lesser extent, in Nhtt16Q-EGFP-expressing cells (Figure 9B). Altogether, these data suggest that downregulation of these modifiers dose not suppress mutant Nhtt toxicity in neuro2a cells.

## Discussion

**Figure 10:**
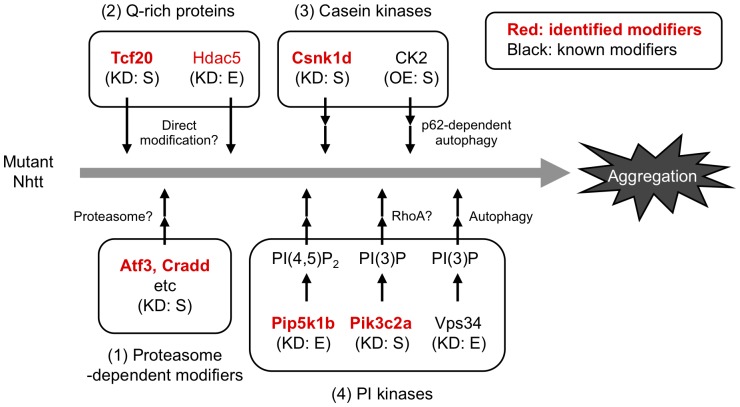
Scheme of modification of mutant Nhtt aggregation by genes identified by shRNA screening. Our experimental data suggest that the genes identified here (indicated by red) modify the mutant Nhtt aggregation by several mechanisms. (1) Proteasome-dependent modification involving Atf3 and Cardd whose knockdown (KD) both suppress the aggregation. (2) Potential direct modification by Q-rich proteins such as Tcf20 and Hdac5, whose knockdown suppresses or enhances the aggregation, respectively. (3) Casein kinase-mediated modification involving Csnk1d whose knockdown suppresses the aggregation. In contrast, overexpression (OE) of CK2 is reported to reduce the aggregates by accelerating p62-dependent autophagic clearance. (4) PI kinase-mediated modification involving Pik3c2a and Pip5k1b, whose knockdown suppresses or enhances the aggregation, respectively. In contrast, knockdown of Vps34, a class III PI3K, is reported to increase the aggregates by attenuating autophagic clearance. The S and E indicate suppression and enhancement of the mutant Nhtt aggregation, respectively.

In this paper, we first performed large-scale shRNA screening of modifiers for mutant Htt aggregation in mammalian cells. By transfection of neuro2a cell line expressing Nhtt with shRNA library clones and automated cell image analysis using ArrayScan HCS reader, we identified 111 shRNAs clones that specifically modified mutant Nhtt aggregation without affecting its expression in neuro2a cells. The shRNA-target genes were classified into various cellular functions including transcription and protein phosphorylation. Subsequent analysis suggests that in addition to the genes such as Atf3 whose knockdown effect was sensitive to proteasome inhibition, there were several genes whose knockdown modified the aggregation independently of it (Figure 10). These include a transcription factor Tcf20 and kinases Csnk1d and Pik3c2a. Notably, all the genes except Pik3c2a [33] are not found by the previous screenings using other organisms such as *Drosophila* and *C. elegans*. Thus, our RNAi screening using mammalian cells identified novel genes that modify mutant Htt aggregation through several, possibly mammalian-specific molecular mechanisms.

An identified modifier, Tcf20 (transcription factor 20), contains several polyQ stretches. Notably, Tcf20, as well as another relatively Q-rich modifier Hdac5 (histone deacetylase 5), was found in mutant Htt aggregates by our previous mass spectrometric analysis [46]. Indeed, we found co-localization and co-insolubilization of Tcf20 with mutant Nhtt aggregates through its N-terminal Q-rich region. In addition, anti-Tcf20 antibody stained nuclear inclusions of R6/2 mouse brain. Furthermore, overexpression of Tcf20 also modified the aggregation. These observations support the idea of physical and probably direct interaction of Tc20 with mutant Htt aggregates, which modifies the aggregation. We have previously shown that an RNA binding protein FUS/TLS, another protein identified by the mass spectrometry described above, suppresses mutant Htt aggregation [8]. In contrast, GIT1 (G protein-coupled receptor kinase-interacting protein), identified by a yeast two-hybrid screen using mutant Htt as a bait, enhances its aggregation [47]. These observations suggest that mutant Htt aggregation is differentially modulated by several interacting proteins, and Tcf20 or Hdac5 may also be the protein that directly modifies it (Figure 10). Interestingly, the modifiers identified here also include several aggregation-prone proteins such as Cradd/Raidd [48], Gpr37/Pael-R [49] and Aimp2/p38 [50]. It would be intriguing to test the co-aggregation of these with mutant Htt, which could lead to identification of another potential mechanism of direct modification of the mutant Htt aggregation.

We also found several kinases that modify the mutant Htt aggregation. One of them is Csnk1d (casein kinase 1 delta; CK1δ), a CK1 family kinase that phoshorylates many substrates with different cellular functions such as cell differentiation, proliferation, chromosome segregation and circadian rhythm [51]. Pathologically, CK1 is shown to be elevated in Alzheimer patients, and phosphorylate tau, a protein linked to Alzheimer's diseases [51]. Although the role of CK1 in polyQ diseases is unknown, overexpression of another casein kinase, CK2, is shown to reduce mutant Htt aggregates possibly through p62-madiated autophagic clearance [36]. Because we found that Csnk1d knockdown reduces the aggregates, mutant Htt aggregation may be differentially modified through these two casein kiasnes, CK1 and CK2 (Figure 10).

Another identified modifier, Pik3c2a (phosphatidylinositol 3 kinase C2 alpha; PI3K-C2α), is a class II PI3K that phosphorylates 3′ position of inositol lipids to produce mainly phosphatidylinositol 3-phosphate (PI(3)P) [52], [53]. Because Pik3c2a knockdown is also shown to reduce mutant Htt aggregation in *Drosophila*, it is an evolutionally conserved modifier of the aggregation. When compared with class I conventional PI3K that produces mainly PI(3,4,5)P_3_ [52], Pik3c2a has unique structural features, including a clathrin-binding site in the N-terminal stretch, and relative resistance to PI3K inhibitors wortmannin and LY294002 [52], [54]. Although recent studies suggest its involvement in intracellular vesicular trafficking and tissue morphogenesis [54]–[57], the mechanism by which Pik3c2a modifies mutant Htt aggregation remains unknown. One potential pathway is through regulation of RhoA small GTPase, because Pik3c2a knockdown impaired RhoA activation in endothelial cell [57], and an inhibitor of Rho kinase, a downstream target of RhoA, suppresses mutant Htt aggregation in neuro2a cells [58]. In contrast, we found that mutant Htt aggregation was enhanced by knocking down another PI kinase, Pip5k1b, which phosphorylates 5′ position of inositol lipids to produce PI(4,5)P_2_. Increased aggregation of mutant Htt has been also reported by suppression of a class III PI3K, Vps34, a key regulator of autophagic clearance of mutant Htt [45]. Thus, several phosphatidylinositols produced by different PI kinases may differentially modulate mutant Htt aggregation possibly through multiple molecular pathways (Figure 10).

Despite the suppression of mutant Nhtt aggregation by knocking down Tcf20, Csnk1d or Pik3c2a, we could not observe clear alteration of mutant Nhtt-induced cell toxicity by it. One possibility is that downregulation of only one gene is insufficient to alter the toxicity. Indeed, functional and/or physical interaction with polyglutamine proteins are also reported for several other shRNA target genes, such as Atf3 [59], Ddx6 [60], Fbxw8 [61], P2ry1 [62] and Map3k1 [63]. In addition, several shRNA targets found in this study are found as modifiers of toxicity in polyglutamine disease models, such as Trappc9 [34], Casp3 [64], Gnf1 (*Drosophila* Rfc1) [65], and F40F8.1 (*C. elegans* Cmpk1) [66]. Notably, gene expression array analysis using R6/2 HD model mice (T.Y and N.N. unpublished data) suggests that some of the shRNA target genes show altered expression in R6/2 brain cortex. These include Rab2b (129% to the control, *P* = 0.0016), Klhl7 (121%, *P* = 0.0050), Fkbp9 (122%, *P* = 0.0098), Plp2 (72.6%, *P* = 0.0023), Mei1 (82.1%, *P* = 0.0208), Pip5k1b (73.6%, *P* = 4.74E-04) and Rassf4 (80.7%, *P* = 4.58E-04). These observations suggest potential *in vivo* significance of these genes for regulation of mutant polyQ aggregation. Further studies are needed to understand the molecular mechanisms underlying regulation of mutant Htt aggregation/toxicity by the modifiers identified in this study.

In summary, through shRNA screening using neuro2a cells, here we identified novel mammalian modifiers of mutant Htt aggregation. Our data suggest that several modifiers mediate their effect possibly through proteasome-independent mechanism such as direct interaction with mutant Htt and protein phosphorylation pathways involving casein kinases and PI kinases. Further studies focused on our identified modifiers may deepen our understanding on the molecular mechanism of mutant Htt aggregation and lead to identification of potential therapeutic targets of HD and other polyglutamine diseases.

## Materials and Methods

### Cell lines

Neuro2a mouse neuroblastome cells, a gift from Dr Iwatsubo (Tokyo University) [35], were maintained in DMEM supplemented with 10% FBS and penicillin-streptomycin in an atmosphere containing 5% CO_2_. Stably transfected neuro2a cells, which inducibly express N-terminal Htt (exon 1) containing 16Q or 150Q fusing EGFP (Nhtt16Q-EGFP or Nhtt150Q-EGFP) under control of ponasterone A, were cultured as above but the medium was further supplemented with 0.4 mg/ml of Zeocin and 0.2 mg/ml of G418 as described previously [35], [46]. Note that Zeocin and G418 were only used for cell maintenance but not for cell transfection experiments described blow.

### Mouse shRNA library

Mouse shRNA libraries (Mouse shRNAmir retroviral library; release 2.3∼2.7 and 2.15) were purchased from Open Biosystems. In this system, shRNAs, cloned in pSHAG-MAGIC2 (pSM2) vector, are expressed under U6 promoter as human microRNA-30 (miR30) primary transcripts and efficiently processed by Drosha and Dicer to produce shRNA. Because the vector contains puromycin resistant gene, the shRNA-expressing cells were able to be selected by treatment the cells with puromycin. The shRNA clones were provided as frozen glycerol stock of E. coli (DH10beta pir116Frt) in 96 well plates. We first amplified the clones in LB medium in 96 well culture plates and purified plasmid DNA by using a plasmid DNA collection system Biomek FX (Beckman Coulter) accompanied with Wizard MagneSil Tfx system (Promega). Clones with no or few growth were excluded at this point. Purities of the clones were estimated by checking their OD260/280 ratio. At least 4∼5 clones in each plate were randomly picked up and subjected to agarose gel electrophoresis to check the DNA integrity.

### shRNA screening using Nhtt-EGFP cell lines

Stable cells for Nhtt16Q-EGFP or Nhtt150Q-EGFP were seeded on 96 well plates in 50 μl of culture medium (10% FBS/DMEM) at density of 2.5×10^4^ cells per well. On the next day, the cells were transfected by adding 15 μl Opti-MEM containing 100 ng of shRNA plasmid DNA and 0.2 μl Lipofectamine 2000 (Invitrogen) to the wells. After 5 hr, 50 μl of medium containing dibutyryl cyclic AMP (dcAMP; final conc. 5 mM) was added to the well. After overnight incubation, the medium was replaced to one containing 2.5 μg/ml puromycin and 5 mM dcAMP for selection of shRNA-expressing cells. After 24 hr incubation, the medium was replaced to one containing 1 μM of ponasterone A and 5 mM dcAMP to induce Nhtt-EGFP expression. After 24 hr, cells were fixed and subjected to nuclear staining with Hoechst or DAPI. The fluorescence cell images in same area of each well were automatically obtained and analyzed by ArrayScan HCS Reader (Cellomics, Thermo Fisher Scientific) along with the protocols designed by the instruction manual. For data analysis of Nhtt150Q-EGFP cells, cells with strong EGFP intensities were counted as aggregate-containing cells because the aggregation accompanied accumulated EGFP signal in the cells. We then divided the number of aggregate-containing cells by total number of cells estimated by counting stained nuclei in the same field, and calculated percentage of cells with aggregates. For data analysis of Nhtt16Q-EGFP cells, EGFP intensities in each cell were quantified and averaged by ArrayScan reader. In both analyses, at least 1000 cells (2000∼3000 cells in average) were analyzed for each well. Scheme of screening procedure is described in Figure 1.

### miRNA construction and gene knockdown

Invitrogen miRNAs (miR RNAi) were designed by BLOCK-iT RNAi Designer in Invitrogen's Web site. Oligo DNAs used for miRNA constructions were listed in Table S1. These were cloned into pcDNA6.2-mRFP-miR vector [36]. By using this vector, transfected cells expressing miRNA were detected by mRFP fluorescence. For gene knockdown, Nhtt150Q-EGFP cells seeded on 24 well plates were transfected with 0.4 μg of miRNA vector by Lipofectamine 2000 (Invitrogen), according to the manufacturer's protocol. After 5 hr of transfection, medium was replaced to one containing 5 mM dcAMP and cells were further incubated for 2 days. Then, the cells were incubated with 1 μM of ponasterone A for 24 hr to induce Nhtt150Q-EGFP expression. The cells were fixed and analyzed by ArrayScan reader and in this case, cells with Nhtt150Q-EGFP aggregates in RFP-positive, miRNA-transfected cells were quantified.

### Treatment of cells with MG132 or Baf A1

Transfection of Nhtt150Q-EGFP cells with shRNA or miRNA was performed as described above. MG132 (Chalbochem) or Baf A1 (LC Laboratories) was co-added to the medium with ponasterone A. After 24 hr incubation, the cells were fixed and cells with aggregates were quantified as above.

### cDNA expression vectors and cell transfection

The mouse cDNAs for full-length Rab2b (clone ID: 4930528G15) and partial regions of Ddr2 (4732470C17) and Tcf20 (4930548B22, I830053D05) were kindly provided as FANTOM3 clones [67]. Remaining regions of Ddr2 and Tcf20 were obtained by RT-PCR to make full-CDS cDNAs. By using Gateway system, these cDNAs were subcloned into the pcDNA-DEST40 vector that expresses cDNA product as C-terminal V5-His tagged protein (Invitrogen). Expression vectors for Tcf20 containing 1∼400 or 1∼500 amino acid were constructed by insertion of their fragments amplified by PCR into pcDNA3.1-V5His vector. Vectors for Nhtt16Q-EGFP, Nhtt150Q-EGFP or Nhtt150Q-EGFP-NLS in pcDNA3.1 vector (Invitrogen) were described previously [35], [46], and pcDNA3.1-LacZ-V5His vector was obtained from Invitrogen. To examine the interaction of the modifiers with mutant Nhtt [12], neuro2a cells were transfected with above expression vectors by Lipofectamine 2000 (Invitrogen) and incubated for one day, For immunofluorescence staining, the cells were fixed with 4% PFA/PBS, permeabilized with 0.1% triton X-100/TBST (20 mM Tris–HCl, pH 8.0, 150 mM NaCl, 0.05% Tween20), and blocked with 5% goat serum/TBST. The cells were then incubated with anti-V5 (R960-25, Invitrogen) diluted with TBST containing 0.1% bovine serum albumin (BSA) for overnight at 4°C, followed by incubation with Alexa 546-conjugated anti-mouse IgG (Molecular Probes). The cells were then mounted in VECTASHIELD with DAPI (VECTOR) and analyzed by Leica confocal system (TCS SP2, SP5). For Western blot analysis, the cells were boiled in SDS sample buffer and subjected to Western blotting as described previously [68]. Primary antibodies used were anti-V5 and anti-GFP (04363, nacalai). Chemiluminescent signals were obtained and quantified using ImageQuant LAS-4000 (GE). To examine the effect of Tcf20 overexpression on mutant Nhtt aggregation, Nhtt150Q-EGFP cells were transfected with expression vectors for Tcf20 (full, 1∼400 or 1∼500)-V5 together with pRFP-C1 vector. The cells were then differentiated by dcAMP on the same day and treated with ponasterone A on the next day. After 24 hr, the cells were fixed and cells with aggregates in trasnfected (RFP-positive) cells were quantified by ArrayScan reader.

### Immunofluorescence staining of mouse brain sections

The mouse experiments were approved by the animal experiment committee at RIKEN Brain Science Institute. Mice were maintained and bred in accordance with RIKEN guidelines. Heterozygous htt exon 1 transgenic male mice of the R6/2 strain were obtained from Jackson Laboratory (Bar Harbor, ME) and maintained as B6CBAF1 background. In this paper, R6/2 male mice and age-matched littermates were used for experiments. For immunofluorescence staining [12], frozen raw brains were cut into 10 μm sections. After fixing with 4% PFA/PBS, the sections were treated with 0.01% H_2_O_2_/methanol at room temperature for 30 min and blocked with 5% skim milk/TBST for 1 hr. The sections were then incubated with a primary antibody diluted with 0.1% BSA/TBST for overnight at 4°C, followed by incubation with a secondary antibody conjugated with Alexa Fluor dyes (Molecular Probes). Primary antibodies used were anti-Htt (EM48; MAB5374, Chemicon), anti-Ub (FK2, BML-PW8810, Enzo) and anti-Tcf20 (sc-86878, Santa Cruz). The tissues were mounted in VECTASHIELD with DAPI and analyzed by a Leica confocal system (TCS SP5).

### Quantitative reverse transcription (RT)-PCR

Preparations of total RNA, reverse transcription and cDNA synthesis from neuro2a cells were performed as described previously [6]. We used following primers designed by Primer Express software (Applied Biosystems) for quantitative PCR; Csnk1d (GCACGCTATGCCTCCATCA, ACCCCAGAGACTCCAAGTCATC), Gapdh (TGTGTCCGTCGTGGATCTGA, CCTGCTTCACCACCTTCTTGA) and Pik3c2a (TTCATAACCTTGCTCAGCTACGTT, GATCCGGCCATCTTGTCTAAAG). Quantitative PCR was performed by SYBR green according to the manufacturer's protocol (Applied Biosystems or Roche). All values obtained were normalized with respect to levels of Gapdh mRNA.

### siRNA transfection and cell toxicity assay

siRNAs were designed based on miRNA target sequences (Table S1) and synthesized by Nippon Gene as 3′ dTdT overhangs. The siRNA sequences without overhangs are follows; no-targeting control (NT) (uagcgacuaaacacaucaa), Tcf20-OBS (gauaucaagucuauuccua), Tcf20-Inv-1 (ccuauaaguguggcgcuuc), Csnk1d-Inv-1 (gcuccuucggagacaucua) and Pik3c2a-Inv-1 (uccugcguuugacauuauu). These were transduced into cells using RNAiMAX (Invitrogen) according to the manufacture's protocol. After one day, the cells were transfected with expression vector for Nhtt16Q-EGFP or Nhtt150Q-EGFP using Lipofectamin 2000 and further incubated for two days in the medium containing 5 mM dcAMP. Then, the cells were co-incubated with 10 μM pyridinium iodide (PI) and 10 μg/ml Hoechst 33342 for 10 min and analyzed by ArrayScan reader. Percent of dead cells in Nhtt-expressing cells was calculated by counting PI-positive cells in the cells positive for both Hoechst and GFP.

### Gene classification

Target genes of final 111 shRNAs were classified using PANTHER Classification System (http://www.pantherdb.org) [37]. Ensembl gene IDs (Data S1) were used as inputs for the analysis. The genes were classified based on their molecular function, biological process, and PANTHER protein class. We also performed Statistical Overrepresentation Test for these genes to examine enrichment of GO term or pathway.

### Statistical analysis

For comparison between two sample groups, data were first analyzed by F-test. For *P*>0.05, the data were analyzed by unpaired Student's t-test (two-tailed); otherwise data were analyzed by Welch's t-test (two-tailed). We considered the difference between comparisons to be significant when *P*<0.05 for all statistical analyses except of third screening in which shRNAs with *P*<0.1 were also included as candidates.

## Supporting Information

Data S1 **Complete list of the screening data for final 111 shRNAs.** Clone IDs, targets genes and data sets of first, second and third screening for final 111 shRNAs are listed. Clone IDs are originally named in this paper based on the plate number and well position of the shRNA. MGI Gene IDs, Ensembl IDs and Entrez Gene IDs in MGI database, and Oligo IDs, Accessions and shRNA sequences in supplemented Open Biosystems (OBS) database are also described.
(XLS)

Data S2 **Sequences of cDNAs used for miRNA construction.** cDNA sequences of shRNA target genes used for miRNA construction. Binding sequences of shRNAs (OBS) are labeled with blue, whereas those of miRNAs (Inv-1/2) are labeled with yellow. Coding DNA Sequences (CDS) are underlined. Note that miRNA-binding sequences are completely different from those of shRNAs except of Gnpda1 in which partial overlap (labeled with green) is observed.
(PDF)

Table S1 **List of the oligos used for miRNA construction.** OBS means the oligos designed based on Open Biosystems shRNA sequence. Inv-1/2 means the oligos designed by BLOCK-iTTM RNAi Designer in Invitrogen's Web site. Number in parenthesis indicates starting position of miRNA-target sequence in coding sequence.
(PDF)
