# Supplementary material for: Large-Scale RNA Interference Screening in Mammalian Cells Identifies Novel Regulators of Mutant Huntingtin Aggregation
Source: PLoS One. 2014 Apr 4;9(4):e93891. doi: 10.1371/journal.pone.0093891 (PMC3976342; doi:10.1371/journal.pone.0093891)
Supplement: Data S2 — Sequences of cDNAs used for miRNA construction. cDNA sequences of shRNA target genes used for miRNA construction. Binding sequences of shRNAs (OBS) are labeled with blue, whereas those of miRNAs (Inv-1/2) are labeled with yellow. Coding DNA Sequences (CDS) are underlined. Note that miRNA-binding sequences are completely different from those of shRNAs except of Gnpda1 in which partial overlap (labeled with green) is observed. (PDF) [file pone.0093891.s002.pdf]

Label
OBS (shRNA): Blue
Inv (miRNA): Yellow
Overlap: Green
CDS Underline

Atf3
CTGGGATTGGTAACCTGGAGTTAAGCGGGCTCCCTGCCAACGCGAGGGCTTTAAAAGGGGTGATGCAACGCGCTCCCAGCCACAG
TCTCACTCAGCGAGACGCCGCGCACGGTGCTTCCCCAGTGGAGCCAATCGGCTAACCCGCGCTCCGGCAGAGTCCTTGGCGCTC
GCCCGCCGGCGGGACAGACCACCCGCCTCTGGCCGCTCTCTGGACCCTGGCCGCCCCGAGCGAAGACTGGAGCAAAATGATGCT
TCAACATCCAGGCCAGGTCTCTGCCTCAGAAGTCAGTGCGACCGCCATTGTCCCCTGCCTCTCACCTCCTGGGTCACTGGTATTTG
AGGATTTTGCTAACCTGACACCCTTTGTCAAGGAAGAGCTGAGATTCGCCATCCAGAATAAACACCTCTGCCATCGGATGTCCTCTG
CGCTGGAGTCAGTTACCGTCAACAACAGACCCCTGGAGATGTCAGTCACCAAGTCTGAGGCGGCCCCTGAAGAAGATGAGAGGAA
AAGGAGGCGGCGAGAAAGAAATAAAATTGCTGCTGCCAAGTGTCGAAACAAGAAAAAGGAGAAGACAGAGTGCCTGCAGAAAGAG
TCAGAGAAACTGGAGAGTGTGAATGCTGAGCTGAAGGCCCAGATTGAGGAGCTGAAGAATGAGAAACAGCATTTGATATACATGCT
CAACCTGCACCGGCCCACCTGCATCGTCCGGGCTCAGAATGGACGGACACCGGAAGACGAGAGGAACCTCTTTATCCAACAGATA
AAAGAAGGAACATTGCAGAGCTAAGCAGAGGTGGCACGGAGGCAATTGGGGAGTTCTTACTGAATCCTCCTTTTCCACCCCACACC
CTGAAGCCATTGGAAAACTGGCTTCCTGTGCACTTCTAGAATCCCAGCAGCCAAGAGCCGTTGGGGCAGGAGGGCCTGTGGTGAC
CTACTGCATTGACCCACTCTGCCCCCGAGTGAACCGTGGAGCAGGCAGGAGCATCCTTTGTCTCACCAATTCCAGGATTTAGGCCT
TATCATCCCGGCCAGTCTCAGATGACCTAGCTGGCCCCAGGCTGGGGTCCTATGCAAAGCAGGATCCCACTAATGGGATTCAGGC
AGAAGTGTCTACCTTGATAGGTGGGGTGGGACCACATCCTCCACTGTGGCTGACAACGCCCTTCCAAGGGAATATGGAATGAGAAC
ATTCATTATTGAGGTTGTCCAATGGCCAGGGTATGCTTTCTAGAAAATATGCTGTTCTGTCCCAGAATGACTGTGCATAGGGTATCC
GTTTCAGAGCCTGGTGTTGTGCTATTTAGATGTTTGTCTTGCACAACATTGGC

Cradd
TTGCCCTCAACAAAGATGGTCTTTATGGTACAGGTTCCCTAGCAGTCTGGATTCCGGTTGTAGTTTTAGTTATTCTTTTTTTTTTTTTT
TTTAAACGGTACGTGGTCGCAGACGAAGAAATGGAAGCCAGAGACAAGCAGGTACTCCGCTCCCTGCGTCTGGAGCTGGGTGCCG
AGGTACTGGTGGAAGGACTGGTTCTTCAGTACCTTTACCAGGAAGGAATTTTGACAGAAAACCACATTCAAGAAATCAAAGCTCAAA
CCACAGGCCTCCGGAAGACAATGCTGTTGCTGGACATCCTGCCTTCCAGGGGCCCCAAAGCTTTTGACACCTTCCTCGATTCCCTC
CAGGAATTTCCCTGGGTAAGAGAGAAGCTGGAGAAGGCGAGAGAGGAAGTCACAGCCGAGCTGCCTACAGGTGACTGGATGGCC
GGAATCCCCTCACACATCCTCAGCAGCTCGCCATCAGACCAGCAGATTAACCAGCTGGCTCAGAGGCTAGGCCCGGAGTGGGAGC
CCGTGGTCCTGTCTCTGGGACTGTCCCAGACCGACATCTACCGCTGCAAGGCCAACCATCCCCACAACGTGCATTCGCAGGTGGT
GGAGGCCTTTGTCCGCTGGCGCCAGCGTTTTGGGAAGCAGGCCACCTTCCTAAGCTTACACAAGGGCCTCCAGGCAGTGGAGGCT
GATCCCTCCCTGCTCCAGCACATGCTGGAGTGACCTGACCCCCCCCGCGCCCCCCCCCCACTTGCTGTGGGGGTGGTGGGGCG
TGGGTTCCCAAGTCACACTGGCTGAACCGGACTTTTCTCAGCAGGTGGCTTTGTTCTGGGCTTTTCAGTGATCTGTTTACGGAAAGA
GATCGTCCACCACTCACTCAACCATCGATTGGCTTTAATTGCTTGAAGACTGCGCTGTTGTAACTATGGTTTGGAACTTTGTGGCTG
GCCTTTAACAGGAGGCCAGAAAAAACACAACACCCACCCTACCCAACCCCCCAAAAAATCATGCTACAGCATCGAATGCAGGTGTC
CTGCATACAAGGCAGCTACACTTGTGTTGCCTGGAGAC

Fbxw8
GGACACTTCCGGCGGATGGACAGTCCGTCCCCATTCTGGCAGCCCCTGGGTGCCGCGTCGGCCTGGGCGCGTCAAGCATGGACG
ACCACAACCTGGAGGAGTTCCGCCGGCACTGGCAGGAGGAGTTGGCGCAGTCACAGGCGCTGAGGCGACGGCGGCGGCTCGAG
GCTGGGGAGCGGCGGTCGCCGCGAAGGCCCGAGGCGGGAGCGCGAGGCGAGCCGGCCTCGGGCTACCTGGGGCTGGCGCAG
GGGCTGCTGGAGGGCGCGGGCCGACCCCCGGCGCCCAGGCCTGGCCGGGGAGGCGACAGGAAGGATACGTCCAGCCGATCGC
GCTCACCCCCAGACCGCGACGCCACGGAGCCCGAGCCGCTGGTGGATCAGCTCATCCGCGACTTGAATGAGCTGGATGACGTGC
CCTTCTTTGATGTCCGTCTGCCTTACGAACTGGCCATCAATATATTCCAGTATCTGAACAGAAGAGAGCTGGGACTGTGTGCTCAGG
TGAGCAAGACATGGAAGGTGATTGCTGAAGACGAGGTGCTGTGGTACCGGCTGTGCCGACAGGAGGGGCACCTTCCCCACAGCA
GATTCTCCGATTACACCTGCTGGAAGCTCATCTTGCAAGAGTGCCTCGCCAAGGAGCACACATTAAGAGCCAACTGGAAGAATCGC
AAAGGTGCCGTGAGCGAGCTGGAGCATGTTCCCGACGCTGTTTTGTGTGACGTGCGTTCTCACGATGGTGTGGTCATTGCTGGATA
CACGTCAGGGGATGTGCGGGTGTGGGACACCCGCACCTGGGACTACGTGGCCCCCTTCCTGGAGTCTGAGTCTGAGGAGGAGGA
TCCTGGAATGCAGCCATATGTCTCCTTTGTGAGGATCAACAGCTCGCTGGCAGTGGCGGCTTACGAGGATGGGATCCTTAACATTT
GGGACCTGAGAACCGGAAGGTTCCCTATCTTTCGTTTTGAGCATGACGCAAGAATACAAGCCCTTGCGCTGAGCCAAGAAAAGCCC
ATTGTTGCCACGGCTTCTGCTTTTGACGTTGTGATGTTGTACCCCAACGAGGAGGGGCATTGGCATGTGGCCTCGGAGTTTGAAGT
TCAGAAGCTGGTTGACTACCTTGAAATAGTTCCGAATACTGGGAGGTACCCTGTGGCAATAGCCACAGCCGGGGATCTGGTGTACC
TGCTGAAGGCCGACGACTCAGCCAGAACCCTTCATTATGTCTATGGCCAGCCTGCCACATGTCTGGATGTCTCAGCCAGCCAGGTT
GCCTTTGGAGTGAAGAGTCTAGGATGGGTGTATGAAGGAAACAAGATCCTGGTGTACAGCCTGGAAGCAGAGCGCTGCCTCTCGA
AGCTGGGCAATGCACTTGGAGACTTTACCTGTGTCAACATCCGGGATAGCCCTCCCAACCTCATGGTCAGCGGCAACATGGACAG
GAGAGTGAGGATCCATGACCTCCGCAGCGATAAGATCGCCCTGTCGCTGTCTGCCCATCAGCTGGGGGTGTCCGCAGTCCAGATG
GATGACTGGAAGGTTGTCAGTGGAGGCGAGGAGGGGCTGGTGTCTGTGTGGGATTACCGCATGAACCAGAAGCTGTGGGAAGTG
CACTCCAGGCACCCTGTGCGCTATCTCTCCTTCAATAGCCACAGCCTCATCACTGCCAACGTGCCCTACGAGAAGGTGCTGCGAAA
CTCCGACCTCGACAACTTTGCCTGTCACAGGAGACATCGTGGCCTGATCCATGCCTATGAATTTGCTGTGGACCAGCTGGCCTTTC
AGAGCCCCCCTTCCTGTCTGCCCGCTTACCCCGTGACATCATGGCTGGATACAGCTATGACCTCGCACTGTCTTTCCCCCATGACAGT
ATTTAGGGTGTCACCTCATGTAGACGTGGAAAGGGCAGTTTTACAAATGTTAGAGTTGGAGAGAGGCTCTGCAGCACATGGTGGGA
GTTTGGGGACAGTGTCCTGTATGACTGTGGCCACACAGCCCTGTTGCCCTGTACAGAACCAGACTCCATTGCTGCCTTTCTCCTCC
TCCTCCTCCTCCTCAGGCTTTGGTAGGACTGGCTGATGACTCAGAGTTAACCTTTCCAGGGGTGGCTCCTCCCCCTCAGCCTATGG
CAGCAGTGACACCCCCCCTCGTTCCATAGGCCAGGGACACAGGGCCTTCACTTGCACTGTCTCCTGGGTGTGGTGCTGAGGGTGG

Gnpda1
GCGCGCACCCCGTGACCGGTGGCCACGTGACGCTCGCGGGCCGGGCCGCTAGTGCTGTAGCTGCTGCAGCCGCGGGAAGCCTG
CGAGTCTAGCGATGAAGCTCATTATCCTGGAACACTATTCCCAGGCCAGTGAGTGGGCGGCCAAGTATATTAGGAACCGTATCATC
CAGTTTAACCCAGGGCCTGACAAGTACTTCACCCTGGGGCTCCCCACTGGGAGCACCCCGCTTGGCTGCTACCAGAAGCTGATCG
AGTACTATAAGAATGGGGACCTGTCCTTTCAATATGTGAAAACCTTCAACATGGACGAGTATGTGGGTCTTCCTCGAGACCACCCAG
AGAGTTACCACTCCTTCATGTGGAATAATTTCTTCAAGCACATTGACATCCACCCTGAAAACACCCACATTTTGGATGGAAATGCGG
CTGACCTGCAGGCCGAGTGTGACGCCTTTGAGGAGAAGATCCAGGCTGCCGGAGGGATCGAACTCTTTGTCGGAGGCATTGGCC
CCGATGGACACATTGCCTTCAATGAGCCAGGCTCCAGCCTGGTGTCCAGGACCCGTGTGAAGACTCTGGCTATGGACACCATCCT

GGCCAACGCTAGGTTCTTTGATGGTGATCTTGCCAAGGTGCCCACCATGGCCCTGACAGTGGGGGTCGGCACTGTCATGGATGCT
AAAGAGGTGATGATCCTCATCACAGGCGCTCACAAGGCCTTTGCTCTGTACAAAGCCATCGAGGAGGGCGTGAACCACATGTGGA
CGGTGTCCGCCTTTCAGCAGCACCCCCGCACTGTGTTTGTGTGTGACGAGGACGCCACCTTGGAACTAAAAGTGAAGACAGTCAAA
TATTTCAAAGGTTTAATGCTTGTTCATAACAAGCTGGTGGACCCCCTGTACAGTATCAAGGAGAAGGAAATTCAGAAAAGCCAATCT
GCGAAGAAGCCATACAGTGACTAGCCTGTGCCAGGACGTGGTATTCAGTACCTCAGAGGGATAGGCAGGTCTTTCCAGAAAGTCTC
TGTAGGAGAGAGAGTAGAATTACTTTTCAGTCCACTCTGGCTGCTGCAGATCTGTGGCTTGGTAGATACATGTTAAGGAGTTTGGCT
ATGGAGAACATTGTTGATTATAATTTTCCCCTTTTTTTTTCAGTACTGGGGGCTGAACCTGGGGCCTTGTACATGCCAAGGAGGTGC
CTTGCTAACTGAGCTATGTCCCCAACTTTCTGCCCCTTTTCATAAT

Pdcd4
CCAGCAGCCCAGCTACCCCAGCTACTCCAGCAGCCCAGCTATCCCAGCTACTCCAGCAGCCCAGCTACCCCAGCTACTCCAGCAG
CCCAGCTACCCCAGCTACCCCAGCTGGGGCTTTTCACAGACGACTAAGTGGGCAGATCCTCAAGGAAGACTTGCATTTAGTAAGTT
GTGTATAATCAGTGCAAGCGAAATTAAGGAAAAATGGATATAGAAAATGAGCAGACACTGAATGTGAACCCCACTGACCCTGACAAT
TTAAGCGACTCTCTCTTTTCTGGAGATGAAGAAAATGCTGGCACTGAAGAAATAAAGAATGAAATAAATGGAAATTGGATTTCCGCAT
CTACTATTAATGAAGCTAGAATCAATGCAAAAGCCAAAAGACGACTGCGGAAAAATTCATCCCGGGACTCTGGCCGAGGAGACTCA
GTCAGTGACAATGGAAGTGAAGCGGTTAGAAGTGGAGTTGCTGTGCCCACCAGTCCAAAAGGAAGGTTGCTAGATAGGCGGTCCA
GATCTGGGAAAGGAAGGGGGCTGCCAAAGAAAGGTGGTGCAGGCGGCAAGGGTGTCTGGGGCACACCTGGACAGGTGTATGATG
TGGAAGAGGTGGATGTGAAAGATCCAAACTATGATGACGACCAGGAGAACTGTGTTTATGAAACTGTAGTTTTGCCCCTGGATGAG
ACCGCATTTGAGAAGACTCTAACACCAATTATACAGGAATACTTTGAGCATGGAGATACAAATGAAGTTGCGGAGATGTTAAGAGAC
TTAAACCTTGGGGAGATGAAGAGTGGCGTGCCGGTGTTGGCAGTGTCCTTAGCCTTGGAGGGGAAGGCCAGCCACCGGGAGATG
ACATCCAAGCTGCTTTCTGACCTTTGCGGGACGGTGATGAGCACAAATGACGTGGAAAAGTCATTTGACAAGTTGCTGAAGGATCT
CCCTGAGCTAGCCTTGGACACTCCTAGGGCACCGCAGTTGGTGGGCCAGTTTATTGCTAGAGCTGTTGGAGATGGAATCTTATGTA
ATACCTATATCGATAGTTACAAAGGAACTGTAGATTGTGTACAGGCTCGAGCTGCTCTGGATAAGGCTACTGTGCTCCTGAGTATGT
CCAAAGGCGGGAAGCGGAAAGACAGTGTGTGGGGATCTGGAGGCGGGCAACAGCCTGTCAATCACCTTGTTAAAGAGATTGATAT
GCTGCTTAAAGAGTATTTACTCTCTGGAGATATATCTGAAGCTGAACACTGCCTTAAGGAACTGGAAGTACCTCATTTTCACCACGA
GCTTGTATATGAAGCCATTGTAATGGTTTTAGAGTCAACTGGAGAAAGTGCATTCAAGATGATCTTAGATTTATTAAAATCCTTGTGG
AAGTCTTCTACTATTACCATAGACCAAATGAAAAGAGGCTATGAGAGAATTTACAATGAAATCCCAGACATTAATCTGGATGTCCCAC
ACTCATACTCTGTTCTTGAGAGATTTGTGGAGGAATGTTTTCAGGCTGGAATAATTTCCAAACAACTCCGTGATCTTTGTCCATCAAG
GGGAAGAAAGCGTTTTGTAAGTGAAGGAGATGGAGGCCGTCTTAAACCTGAGAGCTACTGAGCACAGCAACTCTTACAGTCTTAGG
TGTTACCAAGAACAGATCTCAACTGTAAGAGTTGTCAGTACAGGTTTTCTTTTCCTTTTGTTTTTGTTATTGTTTTTTTTTTTTTTTTTTT
TTTTTTTAAAGAATTTGTTTTGGGTACAAGGCATTTCTAAAATTTTATAAACTTAAGTTTAATGGGATTTTTTGAAGGATTTTCTTTCTTT
TTCTTTTTCTTTCTTTTTTTTTTTTTTTGAGGGGAAATTAATGGAGGGACAGAAGAGGAACCACCGAAGTGTGGGTGTTCTGATAAGCT

Rab2b
GAAGATTTCTGGGGGAGAGCAATAGAGACGCAGCTTGAGCTAGAAGGGCTGGAGCGGAGGCAAATAAGGAGACCCGTAGATTGA
GTGGCTAGAGAGGTCGGAGGTAAGTGGCTGTAGAAATCGGGAGGACCCGGAACACAGACGCCTCGGGACCATGACTTACGCTTAT
CTCTTCAAGTACATCATCATCGGAGACACAGGTGTGGGGAAGTCATGTCTCCTCCTTCAGTTTACCGACAAGCGGTTCCAGCCCGT
GCACGACCTCACAATAGGTGTGGAGTTTGGAGCACGTATGGTCAACATCGATGGAAAACAAATCAAACTGCAAATCTGGGATACGG
CTGGGCAAGAGTCCTTCCGTTCTATCACCCGTTCCTACTACAGGGGAGCAGCTGGAGCCCTGCTGGTGTATGACATCACGAGACG
CGAAACCTTCAACCACCTGACCTCATGGTTAGAGGACGCCCGCCAGCACTCCAGCTCCAACATGGTTATAATGCTGATCGGGAATA
AGAGTGATCTAGAATCTCGAAGGGATGTGAAGAGAGAGGAAGGAGAGGCCTTTGCTCGTGAGCACGGCCTTATATTCATGGAAACA
TCAGCCAAGACAGCCTGCAATGTTGAAGAGGCCTACATTAATACAGCCAAAGAAATATATAGGAAGATCCAGCAAGGTTTATTTGAT
GTCCACAATGAGGCAAATGGCATCAAGATTGGGCCCCAGCAGTCAATTACCTCCTCAGTAGGACCGTGCTCCCCTCAGCAGAACGT
TTCTGACATAGGGCCTGACTCTGGCTGCTGCTGAACACCGGGCCTGAGTGAGCGCTTTCTCTTTCCTCTCGGGGAGCAGCTTCAG
GTCAGCCAGCGCCACGAAGGAGATCCTCGTTGCTTAGGCTGAGAGCAGCCTCTTTTCAGTGTGCGATGTTTCACCCTTCCACTTAA
AACATGAGGAGAAAATTGGGCCCTTTCCGACCTGTCCTCCTTTAGGGACATGAGCATTCCGCGTAGATTATGGCTCTTTCACCCTCT
AGTTTCTAAAATGTTAGAATCAAAGTTGATTGTCACAGTAGCTAAAAGGCAAACAACTTGCTGTCAACATCTATTTGCTAGGTGTTAT

Nap1l4
AGCCCCGGAGCGAGCTTAGGACAGGCGCCACTGCCGCAGGAGGCGCGAGGGGACAAAGGCGTTCAGATGGCAGAAAACAGTCTT
TCAGATGGAGGCCCTGCAGATTCTGTGGAAGCTGCTAAAAATGCCAGTAATACAGAAAAACTCACAGACCAGGTGATGCAGAACCC
ACAAGTGCTGGCAGCTTTGCAGGAACGTCTTGACAACGTCTCTCACACTCCTTCTAGTTACATAGAAACTTTACCTAAAGCTGTCAA
AAGAAGAATTAATGCCCTGAAGCAGCTCCAGGTGAGGTGTGCGCACATAGAAGCTAAGTTCTACGAGGAAGTTCATGACCTGGAGA
GGAAGTATGCAGCCTTGTACCAGCCTCTATTTGACAAGAGAAGAGAATTCATCACTGGTGACGTGGAGCCCACAGATGCAGAGTCA
GCGTGGCACAGTGAGAATGAGGAGGAGGACAAGTTGGCTGGAGATATGAAGAATAAAGTAGTCATAGCTGAGAAAGAAGCAGCAA
CAGTGGAAGAGCTGAACCCCAAAGGCATCCCTGAGTTCTGGTTCACCATCTTCAGAAATGTGGACATGCTTAGTGAGCTGGTGCAG
GAATATGATGAACCGATCTTGAAACATCTGCAGGATATTAAAGTGAAGTTTTCAGACCCTGGACAACCTATGTCTTTTGTGCTAGAGT
TTCACTTTGAACCCAACGACTACTTCACCAATCCTGTCCTGACGAAAACGTACAAGATGAAATCAGAGCCAGACAAGGCCGACCCG
TTTTCTTTTGAAGGTCCTGAAATTGTGGACTGTGATGGGTGTACAATAGACTGGAAGAAAGGAAAAAATGTCACAGTCAAAACCATC
AAGAAAAAGCAGAAGCACAAGGGCCGGGGCACCGTACGAACCATCACCAAGCAAGTGCCCAATGAGTCCTTCTTCAACTTCTTCAG
CCCCCTGAAAGCCTCTGGAGATGGAGAATCTCTGGATGAAGATTCCGAGTTCACCTTAGCCTCTGACTTTGAAATTGGGCACTTTTT
TCGTGAGCGCATCGTGCCACGGGCTGTCCTCTACTTCACTGGGGAGGCCATAGAGGATGATGACAATTTTGAAGAAGGCGAGGAA
GGTGAAGAGGAGGAATTGGAAGGTGATGAAGAAGGAGAAGATGAGGATGATGCTGATGTTAACCCCAAGGTGTAATTTTTGTGTTA
ATCATTCATGCGTTTCTAGAAAGAACCCAGCCAGCCAGCGGAGTGCAAGCAGCAGTGAGAAGCAGTGACCCTGTGGGGCCGCCTT
GTGGATGGCTGTCAGGACCGCAGGCCGCAGGCAGGGGCCTCAGTCCCTGCCAAAGCAGAATTTTGTTGACAGTGCTGTGACATCT
AACCATTTCCAAGTGCATGATTCCTCTCCCCTTTCCCTTCTTACTTTGCTAAACTTGTACAGTGGAGACTGCTGCAGTTCAGAATAGG

Tcf20
GGAGAAACTGCCCCCTTTCCTTTTTCTTCCCGGTTTCTCGCTTTCCCTTCCTCCCTGCCTCCGCGGGGGGTTGTTTTCCTGCCGGC
CTGCATGCTGTGGGCTGTCAAGGGGTTTGGGGGCAAGTGCCAGGGGAGGATGCAGCTGCAAAGGAGGGCTGCTGGCCTGCTGCT
GTGCTGCTGAACAGTATGCAGTCGTTTCGGGAGCAAAGCAGTTACCACGGAAACCAGCAGAGCTACCCACAGGAGGTGCACAGCT
CATCCCGCATAGAAGAGTTCAGCCCTCGCCAGGCCCAGATGTTCCAGAATTTTGGGGGAGCAGGTGGTGGTAGTAGTGGCACTGG
CAGCAGCAGTAGCGGTCGACGAGGAACAGCTGCTGCAGCGGCAGCAATGGCTAGTGAGACCTCTGGCCATCAAGGCTATCAGGG
TTTCAGGAAAGAAGCTGGAGATTTTTACTACATGGCAGGCAACAAAGACACAGTGGCAGCAGGAACCCCACAGCCTCCTCAGCGAA
GGCCTTCTGGGCCTGTACAGAGCTATGGACCTCCCCAGGGGAGCAGCTTTGGCAATCAGTATGCGAGTGAGGGTCATGTGAGCCA
ATTTCAAGCACAGCACTCTGCCCTTGGTGGTGTGTCTCATTATCAGCAGGATTACACAGGGCCTTTCTCTCCTGGGAGTGCTCAGTA
TCAACAGCAGGCCTCTAGCCAACAACAGCAGCAGCAGCAGCAGCAGCAACAGCAGCAGCAGCAGCAACAGCAGCAGCAAGTACA
GCAGTTGAGACAACAGCTTTACCAATCCCATCAGCCTCTGCCACAAACCACTGGACAGCCAGCCTCTGGCTCATCGCATCTACAAC
CAATGCAGCGGCCCTCAACTCTGCCATCTTCTGCTGGTTATCAGTTAAGAGTAGGTCAGTTTGGACAGCACTACCAGTCTTCTGCTT

CCTCCTCTTCCTCCTCCTCCTTCCCTTCTCCACAGCGTTTCAGTCAGTCTGGACAAAGCTATGATGGCAGTTACAGTGTGAATGCTG
GATCTCAGTATGAAGGGCATAATGTGGGTTCTAATGCACAGGCTTATGGAACACAATCAAATTATAGCTATCAACCTCAGTCTATGAA
AAATTTTGAACAGGCAAAGATTCCACCAGGAAACCAGCAGGGGCAGCAGCAGCAACAGCAGCAACCACAACCTCAGCAACAGCAG
CCGCAGCAACAGCAGCAACAGCAGCAGCAGCAGCAACATCCTCCCCAGCATGTGATGCAGTACACAAATGCTGCCACCAAGATGC
CTCTGCAAAGCCAGGTTGGGCAGTACAACCAGCCTGAGGTTCCTGTAAGGTCTCCCATGCAGTTCCACCAGAATTTCAGCCCTATT
TCTAACCCTTCTCCAGCTGCTTCTGTGGTTCAGTCTCCAAGCTGTAGCTCTACACCTTCTCCTCTCATGCAGAGTGGTGAGAATCTC
CAGTGTGGGCAAGGCAATGTGCCCATGAGTTCCAGAAACCGAATTTTACAGCTAATGCCCCAGCTCAGTCCAACTCCATCAATGAT
GCCCAGTCCTAACTCTCATGCTGCAGGATTCAAAGGATTTGGATTAGAAGGGGTGCCGGAAAAGCGGCTGACTGATCCTGGGTTGA
GTAGTTTGAGTGCTTTGAGTAGTCAAGTGGCCAATCTTCCTAATACTGTCCAGCACATGTTACTTTCTGATGCTTTGACACCTCAGAA
GAAGACGTCTAAGAGGCCCTCATCATCATCTAAGAAAGCAGATAGCTGTACAAACTCAGAAGGCTCCTCACAGCCTGAAGAACAAC
TAAAGTCCCCTATGGCTGAGTCATTGGATGGAGGTTGCTCTAGTAGTTCAGAAGATCAAGGTGAGAGGGTGAGGCAACTGAGTGGC
CAGAGCACTAGCTCTGACACCACCTATAAGTGTGGCGCTTCAGAGAAAGCTGGCTCCTCACCAACACAAGGTGCTCAGAATGAGGC
CCCTAGGCTCAGTACCAGTCCTGCAACTAGGGACGAAGCTGCCTCTCCAGGTGCTAAGGACACATCACTGTCATCTGAGGGGAAC
ACAAAAGTCAATGAGAAGACAGTTGGGGTGATTGTCTCCCGAGAAGCCATGACAGGTCGGGTAGAAAAATCTGGTGGACAAGATAA
AGGCTCCCAGGAGGATGATCCTGCTGCCAGTCAGAGACCACCTAGCAATAGTGGCGTAAAGGAAATCAGCCACACATCACTTCCAC
AGCCAGATCCTCCAGGAGGAGGGAGCAAAGGAAACAAGAATGGTGATAATAATAGCTCTAACCACAATGGAGAGGGGAATGGCCC
GAGTAGCCACTCCGCAGTAGGCCCAAGTTTCACAGGCAGGACTGAGCCTAGCAAGTCTCCTGGAAGTTTGCGCTATAGTTACAAAG
AGAGTTTTGGGTCAGCTGTACCACGAAATGTCAGTGGTTATCCTCAGTATCCTTCAGGACAAGAAAAGGGGGATTTTGGCAGCCAT
GGAGAGCGAAAGGGTAGAAATGAGAAGTTCCCAAGTCTTCTACAGGAAGTGCTTCAGGGCTACCACCACCATCCTGACAGAAGGTA
TCCTAGAAGCGCACAGGAACATCAAGGGATGGCTAGTGGCCTGGAAGGAACTGCAAGGCCTAACATCTTAGTCAGTCAAACCAATG
AATTAGCTAGCAGGGGCCTTCTGAACAAGAGTATTGGATCCCTGTTGGAAAATCCCCACTGGGGACCATGGGAAAGGAAGTCAAGC
AGCACAGCTCCTGAAATGAAACAGATCAATTTGTCTGACTATCCCATTCCCAGGAAGTTTGAGATAGAACCTCCATCATCAGCCCAT
GAGCCTGGAGGCTCCCTTTCTGAAAGGAGGTCAGTAATCTGTGATATATCCCCACTAAGACAGATTGTCAGGGACCCAGGGGCTCA
CTCATTGGGACACATGGGTACTGATGCCAGAATTGGGAGGAATGAACGTCTCAACCCAAGTTTAAGTCAGTCAGTCATTCTTCCTGG
TGGGTTGGTGTCCATGGAAACAAAATTGAAATCCCAGAGTGGACAGATAAAAGAGGAAGACTTTGAACAATCCAAATCTCAAGCTAG
TTTCAACAAGAAATCTGGAGACCACTGCCATCCTACCAGCATCAAACATGAGACTTACCGTGGCAATGCAAGTCCTGGAGCAGCAG
CCCATGATTCCATTTCAGACTATGGCCCACAAGATAGCAGGTCCACACCAATGCGGCGGGTCCCTGGTAGAGTTGGTAGCCGGGA
GACTATGAGGGGTCGGTCCTCTTCTCAGTACCATGACTTTGCAGAAAAATTGAAGATGTCTCCAGGCAGGAGCAGAGGCCCAGGG
GGAGACCCTCATCACATGAACCCACATATGACCTTTTCAGAGAGGGCCAATAGGAGTTCTTTACATGCTCCTTTTTCTCCCAACTCA
GAAAGCCTGGCCTCTGCTTACCACACAAACACCAGGGCTCATGCTTATGGGGACCCTAATACCGGTTTGAATTCCCAGCTCCATTAT
AAGAGACAGATGTACCAACAGCAACAAGAGGAGTATAAAGACTGGGCCAGCAGTTCTGCTCAGGGAGTGATCGCTGCTGCGCAGC
ATAGGCAGGAAGGCCCACGCAAGAGCCCAAGGCAGCAGCAGTTTCTTGACAGAGTACGGAGCCCCCTGAAAAATGACAAAGACGG
AATGATGTATGGCCCACCAGTAGGTACGTATCATGACCCAAGCACTCAGGAAGCTGGGCGCTGTCTCATGTCTAGTGATGGTCTGC
CTGCCAAAAGCATGGAATTGAAGCACAGCTCTCAGAAGTTACAAGAGTCTTGTTGGGATCTTTCTCGGCAGACTTCTCCAGCCAAAA
GCAGTGGTCCTCCAGGAATGTCTAATCAAAAACGGTATGGGCCACCCCATGAGCCAGATGGACATGGACTAGCCGAGTCTGCACA
GTCATCCAAACCTAGTAACGTCATGTTACGGCTTCCTGGTCAAGAGGATCATTCTTCTCAAAATCCTTTAATCATGCGAAGGCGGGT
TCGTTCTTTTATCTCCCCTATTCCCAGTAAGAGACAGTCACAAGATGTAAAAAACAGTAACGCTGATGATAAAGGGCGCCTCCTCCA
CCCATCAAAAGAAGGTGCCGATAAAGCATACAATTCCTACAGCCATCTTTCTCACAGTCAGGATATCAAGTCTATTCCTAAGAGAGA
TTCCTCCAAGGACCTTCCAAACCCAGACAATAGAAACTGTCCTGCTGTTACCCTGACAAGTCCTGCTAAGACCAAAATACTGCCCCC
ACGGAAGGGACGGGGATTAAAATTGGAAGCTATAGTTCAGAAGATCACATCTCCAAATATTAGGAGGAGTGCATCTGCAAACAGTG
CTGAGGCTGGGGGAGACACAGTCACACTGGATGATATACTATCTCTAAAGAGTGGTCCTCCTGAAGGTGGCACTGTGGCTACTCAA
GAGGCTGAGATGGAAAAGCGAAAGTGTGAGGTGGTATCTGACCTAGTCAGTGTAACTAACCAGGAATCGAATGTTGAGAAGCCTCT
TCCAGGGCCTTCTGAAGAGTGGCGTGGCAGTGGGGATGACAAAGTCAAGACAGAGGCACATGTAGAAACAGCTTCTACTGGAAAG
GAACCCTCTGGTACTATGACATCCACAGCTTCACAGAAGCCTGGTGGTAACCAAGGGAGACCAGATGGTTCCCTGGGTGGGGCAG
CACCACTAATCTTTCCTGACTCAAAGAATGTAGCTCCAGTGGGCATATTGGCCCCTGAGGCAAATCCCAAGGCTGAAGAGAAAGAG
AACGATACAGTCATGATTTCACCCAAACAAGAAAGTTTCCCCCCTAAAGGGTATTTCCCATCAGGAAAGAAAAAGGGGAGACCAATC
GGTAGTGTCAATAAGCAAAAGAAACAGCAGCAACCACCTCCACCTCCTCAGCCCCCTCAGATGCCAGAAGGTTCTGCAGATGG
GGAGCCAAAGCCAAAAAAGCAGAGGCAAAGGAGGGAGAGAAGGAAGCCTGGGGCCCAGCCAAGGAAGCGGAAAACCAAACAAGC
AGTTCCCATTGTAGAACCTCAAGAACCAGAGATCAAGCTAAAGTACGCTACCCAGCCACTAGATAAAACTGATGCCAAGAACAAGTC
TTTTTTCCCTTACATCCATGTAGTAAATAAGTGTGAACTTGGAGCTGTTTGTACAATCATCAATGCTGAAGAAGAAGAACAGACCAAA
TTGGTGAGGAGCCGGAAGGGTCAGAGATCTCTGACCCCTCCCCCCAGCAGCACAGAAAGCAAGGTGCTCCCAGCTTCATCCTTTA
TGCTGCAGGGGCCTGTGGTAACAGAGTCTTCTGTTATGGGGCACCTGGTTTGCTGTCTCTGTGGCAAGTGGGCCAGTTACCGTAAC
ATGGGTGACCTCTTTGGACCCTTTTATCCCCAAGATTATGCAGCCACTCTTCCGAAGAATCCACCTCCTAAGAGGTCCTCAGAAATG
CAGAGCAAAGTCAAGGTTCGGCACAAAAGCGCTTCTAATGGTTCTAAAACTGACACTGAGGAGGAGGAGGAGCAGCAGCAGCAGA
AGGAGCAGAGGAGCCTGGCTGCTCATCCTAGGTTCAAGCGGCGCCACCGCTCAGAAGACTGTGGTGGAGGTCCTCGGTCCCTGT
CCAGGGGGCTCCCTTGTAAAAAAGCAGCCACGGAGGGCAGCAGTGAAAAGACTGTTTCAGACACAAAGCCCTCTGTACCTACCAC
TTCAGAAGGTGGGCCTGAGCTGGAGTTACAAATCCCTGAACTACCTCTTGACAGCAACGAATTTTGGGTCCATGAGGGTTGTATTCT
CTGGGCCAATGGAATCTACCTGGTCTGTGGCAGGCTCTATGGCCTGCAGGAAGCGCTGGAAATCGCCAGAGAGATGAAATGCTCC
CACTGCCAGGAGGCTGGCGCTACTTTGGGCTGCTACAACAAAGGCTGCTCCTTCCGATACCATTACCCATGTGCCATTGACGCAGA
TTGTTTGCTGCATGAGGAGAATTTCTCGGTGAGGTGCCCCAAGCACAAGCCTCCCCTTCCATGCCCTCTCCCCCCCTTGCAGAACA
AGACCGCAAAAGGCAGCCTCAGCACAGAGCAGTCCGAGCGGGGGTGAGGGGGGAAGTGTGCACGTGGGAATGGACAGCAAGCA
CAGGTGAGACTGTGGAGATGAGAAGGTGGAGGACACACGTGACGGAATGGAATCCTCCTGCTGTGCAACCACACCCCACTCTGCC
CTGCCCTGCCCTGCACCCCCCGCTGCGCTGCGCCTGCCCATGCCAGCACTTCCTTCCTATGTTCTCACATCACACTCAAACTGGTG
ACACCACAGGAAAAGAAAGATACAAGATGTTGGAATGGCTGTTTCCATGGACACAATCTCCATAGTGACAATGTGGGGGAGGGGGG

Tmem179b
GGGCTTGGGTGGTTGTCTTCCTGGTAGCCTGGGCGCCATGGCGCTGCCCTGGTTGCAGCGAGTTGAGCTCTTGCTCTTCACTGCC
GCCTTCCTGTGCGGGGCTTTGGCGGCCGCGACGCTGACCCGGACCCAGGGCTCCTTTGGTGGTAACTGTCCCTTGTATGGTGTG
GCTGCACTCAATGGCTCCTCTCTGGCCTTGTTAGGTCCCTCAGCCCCTTCCCTCTGCTACTTCGTGGCTGGGGCTTCAGGCATCTT
GGCACTCTACTGCCTTCTGCTTCTCTTCTTCTGGGTCTACAGCAGCTGCATCGAAGACTCCCACAGAGGTTCTATAGGGCTCCGAAT
TGCTTTGGCCATCTCAGCTACAGCCATCTTCCTGATCTTAGTGTCTGCCTGTATACTTCGATTTGGCACCAATTCTTTCTGCAATTCC
ATCATCTCCTTGAACCTTACAATTAGCTGCTCTGAAGCCCAGAAAACCTCATGGACACCTTCAGGAACTGCTGTGCAGTTTTACTCC
AACCTACATACTGCTGAAACCTCTTCTTGGGTGAATCTGATATTGTGGTGTCTGGCTTTGCTGCTCCAGGCCATGCAGTGCAAGTTT
AAAGCCACATCATACCAGCCCCAAGAGAGGGGTGACCAAGAGTGGAGCTCTGAGACAGATGCTCTTGTTGGGCACCACCAGTCTC
ACTCCTGAAGAATACTCAAGTGCTTTCTACAACCAGAACCTCCATGCCCCAGTGCTTTTAATCCTCTTGTCTCTGTACAGCCCTGTTT
ATATTAGAAACCTCAGTTTTCTCCTTAAACTTTTTTGTACCAAAATAATATATTACTTTTCTTCATCATGG

Ddr2
GGAAATCTACAAGCGACCTGACATTTGGTGCTCTAGAGCATTCTAAGGCTTGCTGCTTGACTTCTAAAGAAGCTGAAATAATTGAGG

AGGAGCGGGGACCCTCTGTTTCCAAGGACTCTGTTCTGCAGAGAATGTTCTGCACCCTCTGATACTCCAGATCCAACTCCGTCTTC
TGAAATGATCCCGATTCCCAGAATGCCCCTGGTGCTGCTCCTGCTCTTGCTCATCCTGGGTTCTGCAAAAGCTCAGGTTAATCCAG
CCATATGCCGCTATCCTCTGGGCATGTCAGGAGGCCACATTCCAGATGAGGACATCACAGCCTCAAGTCAGTGGTCAGAATCCACG
GCTGCCAAATATGGGAGGCTGGACTCTGAAGAAGGAGATGGAGCCTGGTGTCCTGAGATTCCAGTGCAACCCGATGACCTGAAGG
AATTTCTGCAGATTGACTTGCGAACCCTACACTTTATCACTCTTGTGGGGACCCAGGGGCGCCATGCAGGGGGTCATGGCATTGAA
TTTGCACCCATGTACAAGATCAACTACAGTCGGGATGGCAGTCGCTGGATCTCCTGGCGTAACCGGCATGGGAAGCAGGTGCTTG
ATGGAAACAGTAACCCTTATGATGTATTCCTGAAGGACTTGGAGCCACCCATCGTCGCCAGATTTGTTCGCCTTATCCCAGTCACTG
ACCACTCCATGAACGTGTGCATGAGGGTTGAGCTTTATGGTTGTGTCTGGCTAGATGGCTTGGTATCCTACAATGCTCCAGCTGGA
CAGCAGTTTGTACTCCCTGGAGGCTCCATCATTTATCTGAATGATTCTGTCTATGATGGAGCTGTTGGGTACAGCATGACTGAAGGG
CTAGGCCAGTTGACTGATGGAGTATCCGGCCTGGATGATTTTACCCAGACCCATGAATACCACGTGTGGCCTGGCTATGACTACGT
GGGATGGCGGAACGAAAGTGCTACCAACGGTTTCATTGAGATCATGTTTGAATTTGACCGAATCAGGAATTTTACTACCATGAAGGT
CCACTGCAACAACATGTTTGCTAAAGGTGTGAAGATTTTTAAGGAGGTCCAGTGCTACTTTCGCTCGGAAGCCAGCGAGTGGGAAC
CCACTGCTGTCTACTTTCCCCTGGTCCTGGACGATGTGAACCCCAGTGCCCGGTTTGTCACGGTGCCCCTCCACCACCGAATGGC
CAGTGCCATCAAGTGCCAATACCATTTTGCCGACACGTGGATGATGTTCAGCGAGATCACTTTCCAATCAGATGCTGCAATGTATAA
CAACTCTGGAGCCCTTCCCACCTCTCCTATGGCACCCACCACCTATGATCCCATGCTTAAAGTTGATGATAGCAACACTCGGATCCT
GATTGGTTGCTTGGTGGCCATCATCTTCATCCTGCTGGCTATCATCGTCATCATCCTGTGGAGGCAGTTCTGGCAGAAGATGCTAG
AAAAGGCTTCACGGAGGATGCTGGATGATGAAATGACAGTCAGCCTTTCCCTGCCCAGCGAGTCCAGCATGTTCAATAACAACCGC
TCCTCATCACCAAGTGAACAGGAGTCCAACTCTACTTATGATCGAATCTTCCCCCTTCGCCCTGACTACCAGGAGCCATCCAGACTG
ATCCGAAAGCTTCCAGAGTTTGCTCCAGGAGAGGAGGAGTCAGGGTGCAGTGGTGTTGTGAAGCCGGCCCAGCCCAATGGACCTG
AGGGCGTGCCCCACTATGCAGAAGCCGACATAGTGAATCTCCAGGGAGTGACAGGTGGCAACACCTACTGTGTGCCTGCTGTAAC
CATGGATCTGCTATCGGGGAAAGATGTGGCTGTGGAAGAGTTCCCCAGGAAACTGTTGGCCTTCAAGGAGAAGCTGGGAGAAGGC
CAGTTTGGGGAGGTTCATCTCTGTGAAGTGGAGGGAATGGAAAAATTCAAAGACAAAGATTTTGCACTAGATGTCAGTGCCAACCA
GCCTGTCCTGGTGGCCGTGAAAATGCTCCGAGCAGATGCCAACAAGAATGCCAGGAATGATTTTCTTAAGGAGATCAAGATCATGT
CTCGGCTCAAGGACCCAAACATCATCCGTCTCTTAGCTGTGTGCATCACTGAGGACCCGCTCTGCATGATCACGGAATACATGGAG
AATGGAGATCTTAATCAGTTTCTTTCTCGCCACGAGCCTCTGAGTTCCTGTTCTAGTGATGCCACAGTCAGTTACGCCAACCTGAAG
TTTATGGCAACCCAGATTGCCTCTGGTATGAAGTACCTTTCGTCTCTCAACTTTGTCCACCGAGATCTGGCCACACGAAACTGTTTA
GTGGGCAAGAATTACACCATCAAGATAGCTGATTTTGGCATGAGCAGAAACCTGTACAGTGGTGATTACTACCGGATCCAGGGCCG
GGCGGTGCTCCCCATTCGCTGGATGTCCTGGGAAAGCATCTTGCTGGGCAAATTCACCACGGCAAGTGATGTGTGGGCCTTTGGG
GTGACTCTGTGGGAGACCTTCACCTTTTGCCAGGAGCAGCCCTATTCCCAGCTGTCGGATGAGCAGGTTATCGAGAACACTGGAGA
GTTCTTCCGAGACCAAGGGAGGCAGATCTATCTCCCTCAACCAGCCCTTTGCCCCGACTCTGTGTATAAGCTGATGCTCAGCTGCT
GGAGAAGAGAAACCAAGCACCGGCCATCCTTCCAGGAAATACACCTCCTGCTTCTTCAGCAAGGAGCCGAGTGATGATGCATCAG
CACCTGGCAGTGTTCCTGTGGCCCAGATCCTTCCCACAAGACCTACTGCTCACCCACATCTAAGCCACTCCAGCTGGACATTCAAT
GGACCCGAGAGACAGAGAATTAGTCACTCTGCCCTCTCTGTCCCTTGCCTCCTCCACTACACCTCCCTGCTCCCTACTATTGACTCA
TATATACTTTTTTACATGAAATAACTAAAAGAAGAAAAAAAAAATATAGAGCAGATATTAGTAAAAGAAATGTTACTTGATATACCAAATG

Pik3c2a

GGAAGTGACGTGCAGAGCTCGGCTGGCCGCGGAGTCAGTCGAAGCTCTCCTCAGCGGCCGGCTGAGCCAGCTGAGGCGGGAGA
AAAACATGGCTCGGACCTTGGAGGGCGCGAAGGCTCGGGTTGCGGTGAAGACCAAGACTCCCGCAGCGTGAGGTCCTGGTATTTT
GGAATCTACAAGAAAAAAAGATTAAGAGGTTTGTTCTCTTTTGTGGACATGGCTCAGATTTCCAACAACAGTGAATTTAAACAATGTT
CATCTTCACATCCAGAACCAATAAGAACCAAAGATGTGAACAAAGCAGAAGCGTTACAGATGGAAGCAGAAGCCTTAGCAAAACTG
CAGAAGGATAGACAAATGACTGACAGCCCAAGAGGCTTTGAGCTGTCTAGCAGCACTAGACAAAGAACACAAGGTTTTAACAAACA
GGATTATGATCTCATGGTGTTTCCTGAGTTGGATTCCCAAAAAAGAGCAGTAGATATTGATGTAGAAAAGCTCACCCAGGCTGAACT
TGAGAAGATATTGCTGGACGACAATTTTGAAACTAGAAAACCTCCTGCATTGCCAGTTACTCCTGTTCTGAGCCCTTCGTTCTCAACA
CAGCTGTATCTTAGACCTAGTGGTCAAAGAGGCCAGTGGCCCCCTGGATTATGCGGGCCTTCCACGTACACTTTACCTTCTACTTAT
CCTTCAGCATACAGTAAACAGGCCACATTCCAGAATGGCTTCAGTCCAAGGATGCCCACTTTTCCATCAACAGAGTCTGTATATTTA
AGACTTCCTGGACAGTCTCCATATTTTTCATATCCTTTGACACCTGCCACACCATTTCATCCACAAGGAAGTTTACCAGTCTATCGGC
CACTAGTCAGTCCTGACATGGCAAAACTATTTGAAAAAATAGCAAGTACCTCAGAATTTTTAAAAAATGGGAAAGCAAGGACTGATTT
GGAGATAGCAAACTCGAAAGCTTCAGTCTGCAATCTACAGATATCTCCAAAGTCTGAAGACATCAATAAGTTTGATTGGTTAGACTTG
GATCCTCTGAGTAAGCCTAAGGTAGACTATGTGGAGGTGTTAGAACATGAAGAAGAGAAGAAGGATCCAGTTTTGCTAGCAGAGGA
TCCTTGGGATGCTGTTCTTCTTGAAGAGAGATCGCCAAGTTGTCACCTAGAAAGAAAGGTGAATGGAAAATCCCTTTCTGGGGCAAC
TGTAACAAGAAGCCAGTCTTTAATCATTCGGACAGCTCAATTTACAAAAGCCCAGGGCCAAGTATCTCAGAAAGACCCAAATGGGAC
CAGTAGTTTGCCAACTGGAAGTTCTCTTCTACAAGAATTTGAAGTACAGAATGACGAGGTGGCAGCTTTTTGTCAATCCATTATGAAA
TTGAAGACCAAATTTCCATATACTGATCACTGCACAAATCCAGGCTATTTGTTAAGTCCAGTGACAGTGCAAAGAAACATGTGTGGG
GAGAATGCCAGTGTGAAGGTCTCCATTGAAATTGAAGGGCTTCAACTACCAGTTACTTTTACATGTGATGTGAGTTCTACTGTAGAA
ATAATTATAATGCAAGCCCTTTGCTGGGTACATGATGACTTGAATCAAGTGGATGTTGGCAGCTACATTCTGAAAGTTTGTGGTCAA
GAGGAGGTTCTACAGAATAATCATTGCCTTGGAAGTCACGAACATATTCAAAATTGTCGAAAATGGGACACAGAGATTAAATTACAG
CTCTTGACCTTGAGTGCAATGTGCCAGAATCTGGCTCGAACAGCAGAAGATGATGAAGCACCTGTGGATTTAAACAAATACTTGTAT
CAAATAGAAAAACCTTATAAAGAAGTCATGACAAGACACCCTGTTGAAGAGCTCTTAGATTCCTATCACTACCAAGTAGAACTGGCTC
TTCAAACTGAAAACCAGCACCGAGCTGTTGATCAAGTGATTAAAGCAGTAAGAAAAATTTGTAGTGCTTTAGATGGGGTGGAGACCC
CCTCCGTTACAGAAGCAGTGAAGAAGTTAAAGCGAGCAGTTAACCTTCCAAGGAATAAAAGTGCTGATGTGACTTCATTATCTGGAA
GTGACACAAGGAAGAACTCAACTAAGGGGTCACTGAATCCTGAAAATCCTGTTCAAGTAAGCATGGATCACCTAACAACAGCGATTT
ATGATCTTCTCAGGCTCCATGCAAATTCTAGTAGGTGTTCTACAGGCTGTCCCCGAGGGAGCAGGAACATCAAGGAAGCATGGACT
GCAACGGAGCAGCTCCAGTTCACTGTCTATGCCGCACACGGAATTTCCAGTAACTGGGTATCAAATTATGAAAAATACTACTTGATA
TGTTCCCTGTCTCACAATGGGAAGGATCTTTTTAAGCCTATTCAGTCAAAGAAGGTTGGCACGTACAAGAATTTCTTCTATCTTATTA
AATGGGATGAACTAATCATTTTTCCTATCCAGATATCGCAGTTGCCATTAGAATCAGTTCTTCATCTTACTCTGTTTGGAGTTTTAAAT
CAGAGCAGTGGAAGTTCCCCTGATTCTAATAAACAGAGAAAGGGGCCAGAAGCTCTGGGCAAAGTTTCTTTAACTCTATTTGATTTT
AAACGGTTTTTAACATGTGGAACTAAACTTCTCTACCTTTGGACTTCATCACATACAAATTCTATTCCTGGAGCAATCCCCAAAAAAA
GCTATGTCATGGAAAGAATTGTGCTACAGGTTGATTTTCCTTCTCCTGCGTTTGACATTATTTATACATCTCCTCAAATTGATAGAAAC
ATTATACAGCAAGACAAGTTGGAAACACTGGAGAGTGATATAAAGGGGAAACTTCTGGATATTATTCACAGAGATTCATCATTTGGA
CTTTCTAAAGAAGATAAGGTCTTTTTGTGGGAAAACCGCTATTATTGCCTAAAACATCCAAATTGTCTTCCGAAGATATTAGCAAGTG
CTCCAAACTGGAAGTGGGCTAATCTTGCCAAAACTTACTCATTGCTGCATCAGTGGCCGCCATTGTGCCCACTAGCTGCATTGGAG
CTCCTTGATGCAAAATTTGCTGATCAGGAGGTGCGATCGCTTGCTGTGAGCTGGATGGAGGCCATTAGTGATGATGAGCTAGCAGA
TCTGCTCCCACAGTTCGTACAGGCTTTGAAATATGAAATTTATTTGAATAGTTCACTAGTGCGCTTCCTTCTGTCCAGGGCATTGGGA
AACATCCAGATAGCACACAGTTTGTATTGGCTTCTCAAGGATGCTTTGCATGATACACACTTTGGAAGCAGATATGAACATGTGTTG
GGTGCTCTCCTCTCTGTAGGAGGAAAAGGACTCAGAGAAGAGCTTTCTAAGCAGATGAAACTTGTACAGCTTTTAGGAGGAGTGGC
AGAAAAAGTAAGGCAGGCTAGTGGATCAACAAGACAGGTTGTCCTCCAAAAGAGTATGGAACGGGTACAGTCCTTTTTTCTGAGAA
ATAAATGCCGTCTTCCTCTCAAACCAAGTCTAGTGGCAAAAGAACTAAATATTAAGTCATGTTCGTTCTTCAGTTCTAATGCTATGCC
TCTGAAAGTCACAATGGTGAATGCTGACCCTCTGGGGGAAGAAATTAATGTCATGTTTAAGGTTGGTGAAGATCTTCGGCAAGATAT
GTTAGCTTTACAGATGATAAAGATTATGGATAAGATCTGGCTTAAAGAGGGACTGGATCTGAGGATGGTGATATTCAGATGCCTGTC

AACTGGCCGAGATCGAGGCATGGTGGAGCTAGTTCCTGCTTCAGATACCCTCAGGAAAATCCAAGTGGAATATGGTGTAACAGGAT
CCTTTAAAGATAAACCACTTGCTGAGTGGCTGAGGAAATACAATCCTTCTGAAGAAGAATATGAAAAGGCTTCTGAGAACTTTATCTA
CTCTTGTGCTGGGTGCTGTGTAGCCACCTATGTTTTAGGCATTTGTGATCGGCACAATGACAATATAATGCTTCGAAGCACAGGACA
CATGTTCCACATTGACTTTGGAAAGTTTTTGGGCCATGCACAGATGTTTGGTAGCTTCAAAAGGGACCGAGCTCCTTTTGTGCTTAC
CTCTGACATGGCGTATGTCATTAATGGAGGTGAAAAGCCCACCATTCGTTTCCAGTTGTTTGTGGACCTCTGCTGTCAAGCCTACAA
CTTGATAAGAAAGCAAACAAACCTCTTTCTTAACCTTCTCTCACTGATGATTCCTTCAGGATTGCCAGAACTCACAAGTATTCAGGAT
CTGAAATATGTTAGAGATGCACTTCAGCCCCAAACTACAGATGCTGAAGCTACTATTTTCTTTACTAGGCTGATTGAGTCAAGTTTGG
GAAGCATTGCCACAAAGTTTAATTTCTTCATTCATAACCTTGCTCAGCTACGTTTTTCTGGCCTTCCTTCTAATGATGAGCCCATCCTT
TCATTCTCACCGAAAACATACTCCTTTAGACAAGATGGCCGGATCAAGGAAGTCTCTGTTTTCACATATCATAAGAAATACAACCCAG
ATAAACACTATATTTATGTGGTTCGAATTCTAAGAGAAGGACACCTTGAACCATCATTTGTATTCCGGACATTTGATGAATTTCAGGA
ACTTCACAATAAGCTCAGTATTATTTTTCCTCTTTGGAAATTACCTGGCTTTCCTAATAGGATGGTTCTTGGAAGAACACACATAAAAG
ATGTTGCAGCCAAGAGGAAAATTGAATTAAACAGTTATTTACAGAGTTTGATGAATGCATCAACAGATGTAGCAGAGTGTGATCTTGT
TTGTACTTTTTTCCACCCTTTACTTCGTGATGAGAAAGCTGAAGGAATAGCTAGGTCTGCAGGTGCAGTTCCCTTCAGCCCAACTCT
GGGCCAAATAGGAGGAGCAGTGAAGTTATCTGTTTCTTACCGAAATGGCACCCTCTTCATCATGGTGATGCACATCAAAGATCTTGT
GACTGAAGATGGGGCTGACCCAAATCCCTATGTCAAAACATACCTGCTTCCAGATACCCACAAAACGTCAAAACGTAAAACCAAAAT
TTCACGTAAAACTAGGAACCCAACATTCAATGAAATGCTTGTATATAGTGGATACAGCAAAGAAACTCTGAGGCAGAGAGAACTTCA
ACTGAGTGTACTCAGTGCAGAATCACTGCGGGAGAATTTCTTCTTGGGTGGAATAACCCTGCCACTGAAAGATTTCAACTTGAGCAA
AGAGACAGTTAAGTGGTATCAGCTGACTGCGGCAACGTATCTATAAACTTCCGACTTCTGAGCTTTGGAAACAAGGAGTTATAAATG
TGTGCGCATGCGCACATACACACACTTGGGAACTTTGTATAATTTCATACTTTGGCAGAAATTATAAACTTAACATGTTGGACCAACT
GTCAGTCATATGACTAACTTTTATAGAAGCCATTGCTATTTCAAAGTTATTTTGTTGAGTGCTACCAATCCCAAACTTAATATATACTA
AGTCCTGAAAAAGACTTTGACATAATACTGTGTCATTTTGGTCACCTTGTCTGTACTCTTCCTACACAGACACAGCAAATTGTTTTTTG
TTATGCACCTCACAGCCTTTACCACTGTGTATGTTCACAGACACCAAAGGAAAGATGCAGCTGCTACTCAACAGGCTGAAACGCAAA

Ppt2

ATACGGACCAGGAGTAGGAGCGGAGGCCGGAGAGTTGCTTGCTGTGAGTCTGTTGGGAGTGGAGAGGCGTCGTCTCCCTCACGG
CCTCTCGTTCCTTCTCAGGTGGGAGCATGCCGGGGCTATGGAGGCAGAGGCTTCCTTCGGCTTGGGCTTTGCTTCTCCTGCCGTT
CCTGCCGCTGCTGATGCCCGCAGCCCCCGCAGCCCACCGCGGGTCCTACAAGCCCGTGATCGTGGTGCACGGGCTCTTTGACAG
TTCATACAGCTTCCGCCACCTGCTGGACTATATCAATGAGACACACACCGGGACTGTGGTGACAGTGCTTGATCTCTTCGATGGCA
GAGAGAGTTTGCGGCCCCTGTGGGAACAGGTACAAGGGTTCCGAGAGGCTGTGGTCCCCATCATGGAAAAGGCCCCTGAAGGAG
TGCACCTCATCTGCTACTCCCAGGGGGGCCTGGTGTGCCGTGCTTTGCTGTCTGTCATGGATAACCACAATGTGGACTCTTTCATC
TCCCTCTCCTCCCCACAGATGGCCAGTATGGAGACACGGACTATTTGAAATGGCTCTTCCCCACGTCCATGCGGTCTAACCTCTA
TCGGGTCTGCTATAGTCCTTGGGGCCAGGAATTTTCCATTTGCAACTACTGGCACGATCCTCACCACGATGACTTGTACCTCAATGC
CAGCAGCTTTCTGGCCCTCATCAATGGGGAAAGAGACCATCCCAATGCCACTGCATGGCGGAAGAACTTCCTTCGCGTGGGCCGT
CTGGTGCTGATTGGGGGTCCTGATGATGGCGTTATCACTCCCTGGCAATCTAGCTTCTTTGGTTTCTATGATGCCAATGAGACAGTT
CTGGAGATGGAGGAGCAGCCGGTGTATCTTCGAGATTCTTTTGGGTTGAAGACTCTCCTGGCCCGGGGGGCCATAGTGAGGTGTC
CCATGGCTGGCATCTCTCACACCACGTGGCACTCTAACCGTACGCTCTACGATACTTGCATTGAGCCGTGGCTCTCCTGAAGATGT
CCTCAGTGGTCTCCAGGAATTCCCCAGCCCAGAGATCAAGCGGTGGCCTTGGGAAGCAGATGCCAGGCTCTGGCATGCCTGCAAC
CATCTTGTCATTCCCACACTGCCCGACCCCCACCAGGGCTCCCCTCCCCCCTCCCCCCCTGCTCCTCTGTGAATGACAAGTCCTGG
TCCCCTACCTCATGTCCTCACTTGGGGACGCCTCCACGCTCTCCCTTTCTGCCACGGCTGAGGTTGGGAAGCAAGTGCCAGGTTTT
TAACTGTGGCTTCACCGCTGCTGTTGCTTCTCCGGGTCTGGCTGTATCGGAGGAGAACCCAGCCCCTGCCCACCTCAGGGGTCTT
CTTCCAGGCCACTCAGGACATTTTTAGCTTCTGTTATCCCCGTTTCCCTCTTTTCTCAACTTCCCTGTGGCTAGCCTCCCCACCAAGA
GGGGCACCCATAAGAGGGGGTGTTCTGAGGCTCCCCTATGGGGACAATTCCACTTTTGAAGTGTCAGTGTTGGGGAATATCTGTG
GCCTGCAAGGCCCATCTCAGGTTTGGGGATCCCCCAGTCCCTATGATCAGTGTTGGGGTATCCCCTGGGAGCCTAGGTTCTTTGA
GGCCCCAGCCCCTCTTTTAACTACCCTTGAGTGGGTGGTCCCTGTATTTATAGAAATAAAAGATCCATTTCCTCAG

Csnk1d

GAGGGCGAGCGGGGCCGGCAAGGGCCGGAGCAGCGGCGGCGGCGCTCGGACTGTCCCATCCGCCCCGTATTGAGGCGCTGAG
AGCGACGGGGCGACCGGAAAGCGATGGTGAAAGCGGGGCCGTGAGGGGGGCGGAGTCGGACCGGACCCGCAGTAGCGGCAGC
AGCGGCGCCGCCTCCCGGAGCGCAGACCCAGGAAGCGGCCGGGCGGGCAGTAGCGAGCCGCACCGCTGCCATGGAGCTGAGG
GTCGGGAACAGGTACCGACTGGGCCGCAAGATCGGCAGCGGCTCCTTCGGAGACATCTATCTCGGTACGGACATTGCTGCGGGA
GAAGAAGTTGCCATCAAGCTTGAATGTGTCAAAACCAAACATCCTCAGCTCCACATTGAGAGCAAGATCTACAAAATGATGCAGGGA
GGAGTGGGCATCCCTACCATCAGATGGTGTGGGGCTGAGGGGGACTACAATGTCATGGTGATGGAGCTACTGGGACCCAGCCTG
GAAGACCTGTTCAACTTCTGTTCAAGGAAGTTTAGTCTCAAAACTGTTCTGTTGCTTGCTGACCAAATGATAAGTCGTATTGAGTACA
TTCATTCGAAGAATTTTATCCACCGAGATGTGAAGCCAGATAACTTCCTCATGGGGCTGGGAAAGAAAGGCAACCTGGTCTACATCA
TTGACTTTGGGCTGGCCAAGAAGTATCGGGATGCACGCACCCACCAGCATATCCCCTATCGAGAGAACAAGAACCTCACAGGGAC
AGCACGCTATGCCTCCATCAACACGCACCTTGGCATTGAACAATCTCGAAGGGATGACTTGGAGTCTCTGGGGTACGTGCTGATGT
ACTTCAACCTGGGCTCTCTCCCCTGGCAGGGGCTGAAGGCCGCCACCAAGAGGCAGAAGTATGAGAGGATCAGTGAGAAGAAGAT
GTCCACTCCCATTGAAGTGCTGTGCAAGGGCTATCCTTCTGAATTTGCCACATACCTGAATTTCTGCCGTTCCTTACGTTTTGATGAC
AAACCTGACTACTCCTACCTGAGACAGCTCTTCAGAAATCTGTTCCATCGCCAAGGCTTCTCCTACGACTATGTGTTCGACTGGAAC
ATGCTCAAATTTGGTGCCAGCCGGGCTGCAGATGATGCTGAGCGGGAACGCCGGGATCGAGAAGAACGATTAAGACACTCCCGGA
ATCCAGCCACTCGTGGCCTCCCTTCTACAGCTTCCGGCCGTCTGCGGGGAACCCAGGAAGTGGCTCCCCCAACGCCCCTTACCCC
TACCTCACACACGGCCAACACCTCTCCGAGGCCTGTCTCTGGCATGGAACGAGAACGGAAAGTGAGTATGCGGCTGCACCGTGGG
GCCCCAGTCAACGTCTCCTCATCTGATCTCACGGGCCGACAAGATACCTCTCGCATGTCCACCTCACAGATTCCCGGTCGGGTGG
CTTCCAGTGGTCTTCAGTCTGTCGTGCACCGATGAGAACTCTCCTTTTTGCTGTGAAGGGCAGACAATGCATGGCTGATCTACTCTG
TTACCAATGGCTTTACTAGTGACACGCCCCCCGGTCTAAACCTGAAATGTTAACGCCGGGAGCTCTCCAGGCCACTCACCCAGCGA
TGCACATGGGGAATCAAAACACAAACTAAATGGACAGGCTCCAAGAGCTGCCATCGCCAGGGGCTGCCACCGTGGCCCCGTGTGA
GCTCAGTTATCATGGGGCTGGGACGAAGAGCAGAGGCTGGGAGACCATTGCAGAGAGAACCCGACTCCCTGTGAA

Map3k1

GCCCGCGAGAGAAATGGCGGCGGCGGCGGGCGATCGCGCCTCGTCGTCGGGATTCCCGGGCGCCGCGGCGGCGAGTCCCGA
GGCGGGCGGCGGCGGCGGAGGAGGAGGAGCTCTCCAGGGAAGCGGCGCGCCCGCAGCGGGCGCGGCGGGGCTGCTGCGGGA
GCCTGGCAGCGCGGGCCGCGAGCGCGCGGACTGGCGGCGGCGGCAGCTGCGCAAAGTGCGGAGTGTGGAGCTGGACCAGCTG
CCGGAGCAGCCGCTCTTCCTCGCCGCCGCCTCGCCGCCCTGCCCATCTACTTCCCCGTCGCCGGAGCCCGCGGACGCGGCTGCA
GGAGCGAGTCGCTTCCAGCCCGCGGCGGGACCGCCACCCCCGGGAGCGGCGAGTCGCTGCGGCTCCCACTCTGCCGAGCTGGC
GGCCGCGCGGGACAGCGGCGCCCGGAGCCCCGCGGGGGCCGAGCCGCCCTCTGCAGCGGCCCCCTCCGGTCGAGAGATGGAG
AATAAAGAAACCCTCAAAGGACTGCACAAGATGGAGGATCGCCCGGAGGAGAGAATGATCCGGGAGAAGCTCAAGGCGACCTGTA
TGCCCGCCTGGAAGCACGAGTGGTTGGAGAGGAGGAACAGGAGAGGCCCTGTGGTGGTGAAGCCAATCCCTATTAAAGGAGATG
GATCTGAAATGAATAACTTGGCAGCTGAGCCCCAAGGAGAGGGCCAGGCAGGTTCCGCTGCACCAGCCCCCAAGGGCCGACGAA
GCCCATCTCCTGGCAGCTCTCCGTCAGGGCGCTCGGTGAAGCCGGAATCCCCAGGAGTGAGACGGAAACGAGTGTCCCCGGTGC

CTTTCCAGAGTGGCAGAATCACACCACCCCGAAGAGCCCCGTCACCGGATGGCTTCTCCCCGTACAGCCCAGAGGAGACGAGCC
GCCGCGTGAACAAAGTGATGAGAGCCAGGCTGTACCTGCTGCAGCAGATAGGACCCAACTCTTTCCTGATTGGAGGAGACAGTCC
AGACAATAAATACCGGGTGTTTATTGGGCCACAGAACTGCAGCTGTGGGCGTGGAGCATTCTGTATTCACCTCTTGTTTGTCATGCT
CCGGGTGTTTCAGCTAGAACCCTCTGACCCCATGTTATGGAGAAAAACTTTAAAAAATTTCGAGGTTGAGAGTTTGTTCCAGAAATA
CCACAGTAGGCGTAGCTCGAGAATCAAAGCTCCATCCCGGAACACCATCCAGAAGTTTGTGTCACGCATGTCAAATTGTCACACAC
TGTCATCGTCTAGCACATCCACATCTAGTTCAGAAAACAGCATCAAGGATGAAGAGGAGCAGATGTGTCCCATCTGCTTGCTGGGC
ATGCTGGATGAGGAGAGCCTGACTGTGTGTGAAGATGGCTGCAGGAACAAGCTGCACCACCATTGCATGTCCATCTGGGCGGAAG
AGTGTAGAAGAAATAGAGAGCCTTTAATATGTCCCCTTTGTAGATCTAAGTGGAGATCCCATGACTTCTACAGCCATGAGTTATCAA
GCCCCGTGGAGTCCCCCACCTCCCTGCGAGCTGTCCAGCAGCCATCCTCCCCACAGCAGCCCGTGGCCGGATCACAGCGAAGGA
ATCAGGAGAGCAGTTTTAACCTTACTCATTTTGGAACCCAGCAGATTCCTTCCGCTTACAAAGATTTGGCCGAGCCATGGATTCAGG
TGTTTGGAATGGAACTCGTTGGCTGCTTATTCTCTAGAAACTGGAACGTAAGGGAAATGGCCCTTAGGCGTCTTTCCCACGACGTTA
GTGGGGCCCTGTTGTTGGCAAACGGGGAGAGCACTGGAAACTCTGGAGGCGGCAGTGGGGGCAGCTTAAGCGCGGGAGCGGCC
AGCGGGTCCTCCCAGCCCAGCATCTCAGGGGATGTGGTGGAGGCGTGCTGCAGTGTCCTGTCTATAGTCTGCGCTGACCCTGTCT
ACAAAGTGTACGTTGCTGCTTTAAAAACATTGAGAGCCATGCTGGTATACACTCCTTGCCACAGTCTGGCAGAAAGAATCAAACTTC
AGAGACTCCTCCGGCCAGTTGTAGACACTATCCTTGTCAAGTGTGCAGATGCCAACAGCCGCACGAGTCAGCTGTCCATATCTACA
GTGCTGGAACTCTGCAAGGGCCAAGCAGGAGAGCTGGCGGTTGGGAGAGAAATACTTAAAGCTGGGTCCATCGGGGTTGGTGGT
GTCGATTACGTCTTAAGTTGTATCCTTGGAAACCAAGCTGAATCAAACAACTGGCAAGAACTGCTGGGTCGCCTCTGTCTTATAGAC
AGGTTGCTGTTGGAATTTCCTGCTGAATTCTATCCTCATATTGTCAGTACTGATGTCTCACAAGCTGAGCCTGTTGAAATCAGGTACA
AGAAGCTGCTCTCCCTCTTAACCTTTGCCTTGCAATCCATTGACAATTCCCACTCGATGGTTGGCAAGCTCTCTCGGAGGATATATC
TGAGCTCTGCCAGGATGGTGACCGCAGTGCCCGCTGTGTTTTCCAAGCTGGTAACCATGCTTAATGCTTCTGGCTCCACCCACTTC
ACCAGGATGCGCCGGCGTCTGATGGCTATCGCGGATGAGGTAGAAATTGCCGAGGTCATCCAGCTGGGTGTGGAGGACACTGTG
GATGGGCATCAGGACAGCTTACAGGTGCTGGCCCCCGCCAGCTGTCTAGAAAACAGCTCCCTTGAGCACACAGTCCATAGAGAGA
AAACTGGAAAAGGACTAAGTGCTACGAGACTGAGTGCCAGCTCGGAGGACATTTCTGACAGACTGGCCGGCGTCTCTGTAGGACT
TCCCAGCTCAACAACAACAGAACAACCAAAGCCAGCGGTTCAAACAAAAGGCAGACCCCACAGTCAGTGTTTGAACTCCTCCCCTT
TGTCTCATGCTCAATTAATGTTCCCAGCACCATCAGCCCCTTGTTCCTCTGCCCCGTCTGTCCCAGATATTTCTAAGCACAGACCCC
AGGCATTTGTTCCCTGCAAAATACCTTCCGCATCTCCTCAGACACAGCGCAAGTTCTCTCTACAATTCCAGAGGAACTGCTCTGAAC
ACCGAGACTCAGACCAGCTCTCCCCAGTCTTCACTCAGTCAAGGCCCCCACCCTCCAGTAACATACACAGGCCAAAGCCATCCCGA
CCCGTTCCGGGCAGTACAAGCAAACTAGGGGACGCCACAAAAAGTAGCATGACACTTGATCTGGGCAGTGCTTCCAGGTGTGACG
ACAGCTTTGGCGGCGGCGGCAACAGTGGCAACGCCGTCATACCCAGCGAAGAGACAGTGTTCACGCCGGTGGAGGACAAGTGCA
GGTTAGATGTGAACACCGAGCTCAACTCCAGCATCGAGGACCTTCTTGAAGCATCCATGCCTTCAAGTGACACGACAGTCACTTTC
AAGTCCGAAGTCGCCGTCCTCTCTCCGGAAAAGGCCGAAAATGACGACACCTACAAAGACGACGTCAATCATAATCAAAAGTGCAA
AGAAAAGATGGAAGCTGAAGAGGAGGAGGCTTTAGCGATCGCCATGGCGATGTCAGCGTCTCAGGATGCCCTCCCCATCGTCCCT
CAGCTGCAGGTGGAAAATGGAGAAGATATTATCATCATTCAGCAGGACACACCAGAAACTCTTCCAGGACATACCAAAGCGAAACA
GCCTTACAGAGAAGACGCTGAGTGGCTGAAAGGCCAGCAGATAGGCCTCGGAGCATTTTCTTCCTGTTACCAAGCACAGGATGTG
GGGACTGGGACTTTAATGGCTGTGAAACAGGTGACGTACGTCAGAAACACATCCTCCGAGCAGGAGGAGGTGGTGGAAGCGTTGA
GGGAAGAGATCCGGATGATGGGTCACCTCAACCATCCAAACATCATCCGGATGCTGGGGGCCACGTGCGAGAAGAGCAACTACAA
CCTCTTCATTGAGTGGATGGCGGGAGGATCTGTGGCTCACCTCTTGAGTAAATACGGAGCTTTCAAGGAGTCAGTCGTCATTAACT
ACACTGAGCAGTTACTCCGTGGCCTTTCCTATCTCCACGAGAACCAGATCATTCACAGAGACGTCAAAGGTGCCAACCTGCTCATT
GACAGCACCGGTCAGAGGCTGAGAATTGCAGACTTTGGAGCTGCTGCCAGGTTGGCATCAAAAGGAACCGGTGCAGGAGAGTTCC
AGGGACAGTTACTGGGGACAATTGCATTCATGGCGCCTGAGGTCCTAAGAGGTCAGCAGTATGGTAGGAGCTGTGATGTATGGAG
TGTTGGCTGCGCCATTATAGAAATGGCTTGTGCAAAACCACCTTGGAATGCAGAAAAACACTCCAATCATCTCGCCTTGATATTTAA
GATTGCTAGCGCAACTACTGCACCGTCCATCCCGTCACACCTGTCCCCGGGTCTGCGCGACGTGGCGCTGCGCTGCTTAGAACTT
CAGCCTCAGGACCGGCCTCCGTCCAGAGAGCTGCTGAAACATCCGGTCTTCCGTACCACGTGGTAGTTAATTGTTCAGATCAGCTC
TAATGGAGACAGGATATGCAACGCGGAGAGAGAAAAGAGAACTTGTGGGCGACCATGCCGCTAACCCGCAGGCCCTCACGCCACT
GAACAGCCAGAAACGGGGCCAGCGGGGAACCGTACCTAAGCATGTGATTGACAAATCATGACCTGTACCTAAGCTCGATATGCAG
ACATCTACAGCTCGTGCAGGAACTGCACACCGTGCCTTT

Pip5k1b

CCTGGGGGAACAAAAGCAGTGTCAACGATGAGCAGACCACCCGTCACCACCAACTCCAGATGACTTGCCATTTTCCTTACAAAGAT
GTCGTCAACTGCTGAAAATGGAGACGCCGTACCTGGAAAACAAAACGAAGAAAAAACCTACAAAAAGACGGCATCGTCAGCTATTA
AAGGTGCTATCCAGCTGGGAATAGGATACACAGTGGGCAACCTCACGTCCAAGCCAGAACGCGATGTCCTCATGCAAGACTTCTAC
GTGGTGGAAAGTGTGTTCCTCCCCAGTGAAGGGAGCAACCTGACCCCAGCACATCACTACCCAGACTTCAGGTTTAAGACCTACGC
ACCGCTGGCCTTCCGGTACTTCCGAGAACTCTTTGGTATCAAGCCCGATGATTACTTGTACTCCATCTGCAGTGAACCTCTGATAGA
ACTGTCCAATCCTGGAGCCAGTGGGTCCTTGTTTTTCTTGACCAGCGATGATGAATTTATCATCAAAACCGTTCAGCATAAGGAAGC
CGAGTTCCTGCAGAAGCTGCTGCCGGGCTATTACATGAATTTAAACCAGAATCCAAGGACTCTTCTGCCAAAATTCTATGGGCTGTA
TTGCATGCAGTCAGGAGGCATCAACATCCGTATTGTGGTGATGAACAATGTCCTGCCGCGTGCCATGAGGATGCATTTGACCTATG
ACCTGAAAGGCTCCACATACAAGCGAAGAGCATCCCGAAAAGAGAGGGAGAAACCCAACCCCACGTTTAAGGACCTGGACTTCCT
GCAGGACATGCACGAGGGGCTGTATTTCGATACAGAAACATACAATGCCCTTATGAAGACACTACAGAGAGACTGCCGGGTTCTGG
AAAGCTTCAAGATCATGGACTACAGCCTTTTGCTGGGTATCCACATCTTGGACCATTCCCTCAAAGACAAAGAAGAGGAGCCCCTCC
AAAACGTGCCTGATGCAAAGCGGCCGGGGATGCAGAAAGTCCTCTATTCCACAGCCATGGAATCCATCCAGGGTCCAGGCAAGTC
TGCAGATGGGATCATTGCAGAGAATCCAGACACAATGGGAGGCATTCCAGCTAAAAGCCATAAGGGAGAGAAGCTGCTTCTATTCA
TGGGCATTATTGACATCCTCCAATCATATAGGTTAATGAAGAAGTTAGAACATTCCTGGAAAGCCCTTGTTTATGATGGGGACACCG
TTTCAGTTCATAGACCAAGTTTTTATGCAGATAGATTTTTAAAGTTTATGAATTCCAGAGTTTTCAAGAAAATTCAAGCTCTGAAAGCC
TCGCCTTCTAAGAAACGCTGCAACTCCATCGCTGCACTGAAGGCAACCTCCCAGGAGATCGTATCCTCCATCAGCCAGGAATGGAA
GGACGAGAAGCGGGATTTGTTAACCGAAGGACAGAGTTTCAGCAGCCTGGATGAAGAAGCCCTGGGATCCCGACACAGGCCCGA
CCTGGTACCCAGCACTCCATCATTATTTGAAGCTGCTTCCTTAGCGACCACAATATCATCTTCTTCCTTATATGTCGGCGAACACTAT
CCACATGACAGGACTACCCTTTACTCAAACAGCAAAGGGTTACCTTCCAGTTCAACCTTCACCTTGGAAGAGGGGACCATCTACCTG
ACGGCTGAGCCAAACACCCTGGACCTGCAGGATGATGCCTCTGTGCTGGACGTCTATTTATAAGTGAAAGCGGCAGCCACCTGAG
CACGTGAGCGGGACCTGAACACAGCCATGGGAGGGAACTTCCCCCACTGCAGAATTCCTCCAGAAGAACTGGGCTGAACCCCAGC
ACACACAGTAAGACTCAGCTGGACCTTGAAGCCCGCAAGATGCCGGGGAGCCTCGGTCCGTGGCCGATCCGCAGATGGATGTGA
AAATCCCACACCGTTCCGTGGGTGCTGCTGCTTGCTGACCATGAAGACCTGCATGACCTCTGTCTAAGCCGCTTTTCTACACCATG
