# Supplementary material for: Large-Scale RNA Interference Screening in Mammalian Cells Identifies Novel Regulators of Mutant Huntingtin Aggregation
Source: PLoS One. 2014 Apr 4;9(4):e93891. doi: 10.1371/journal.pone.0093891 (PMC3976342; doi:10.1371/journal.pone.0093891)
Supplement: Table S1 — List of the oligos used for miRNA construction. OBS means the oligos designed based on Open Biosystems shRNA sequence. Inv-1/2 means the oligos designed by BLOCK-iTTM RNAi Designer in Invitrogen's Web site. Number in parenthesis indicates starting position of miRNA-target sequence in coding sequence. (PDF) [file pone.0093891.s003.pdf]

Gene	miRNA	OligoDNA	Sequence
Atf3	Inv-1 (117)	Top	TGCTGCTCAGCTCTTCCTTGACAAAGGTTTTGGCCACTGACTGACCTTTGTCAGAAGAGCTGAG
		Bottom	CCTGCTCAGCTCTTCTGACAAAGGTCAGTCAGTGGCCAAAACCTTTGTCAAGGAAGAGCTGAGC
	Inv-2 (148)	Top	TGCTGATGGCAGAGGTGTTTATTCTGGTTTTGGCCACTGACTGACCAGAATAAACCTCTGCCAT
		Bottom	CCTGATGGCAGAGGTTTATTCTGGTCAGTCAGTGGCCAAAACCAGAATAAACACCTCTGCCATC
Cmpk1	OBS	Top	TGCTGATTGGTTTCGTTGATTCAAGGGTTTTGGCCACTGACTGACCCTTGAATACGAAACCAAT
		Bottom	CCTGATTGGTTTCGTATTCAAGGGTCAGTCAGTGGCCAAAACCCTTGAATCAACGAAACCAATC
Cradd	Inv-1 (6)	Top	TGCTGTTCCTGGAGGGAATCGAGGAAGTTTTGGCCACTGACTGACTTCCTCGACCCTCCAGGAA
		Bottom	CCTGTTCCTGGAGGGTCGAGGAAGTCAGTCAGTGGCCAAAACTTCCTCGATTCCCTCCAGGAAC
	Inv-2 (524)	Top	TGCTGTGTGTAAGCTTAGGAAGGTGGGTTTTGGCCACTGACTGACCCACCTTCAAGCTTACACA
		Bottom	CCTGTGTGTAAGCTTGAAGGTGGGTCAGTCAGTGGCCAAAACCCACCTTCCTAAGCTTACACAC
Csnk1d	OBS	Top	TGCTGTTAACATTTCAGGTTTAGACCGTTTTGGCCACTGACTGACGGTCTAAATGAAATGTTAA
		Bottom	CCTGTTAACATTTCATTTAGACCGTCAGTCAGTGGCCAAAACGGTCTAAACCTGAAATGTTAAC
	Inv-1 (52)	Top	TGCTGATAGATGTCTCCGAAGGAGCCGTTTTGGCCACTGACTGACGGCTCCTTGAGACATCTAT
		Bottom	CCTGATAGATGTCTCAAGGAGCCGTCAGTCAGTGGCCAAAACGGCTCCTTCGGAGACATCTATC
	Inv-2 (272)	Top	TGCTGTTGAACAGAAGTTGAACAGGTGTTTTGGCCACTGACTGACACCTGTTCCTTCTGTTCAA
		Bottom	CCTGTTGAACAGAAGGAACAGGTGTCAGTCAGTGGCCAAAACACCTGTTCAACTTCTGTTCAAC
Ddr2	Inv-1 (178)	Top	TGCTGTTCAGAGTCCAGCCTCCCATAGTTTTGGCCACTGACTGACTATGGGAGTGGACTCTGAA
		Bottom	CCTGTTCAGAGTCCACTCCCATAGTCAGTCAGTGGCCAAAACTATGGGAGGCTGGACTCTGAAC
	Inv-2 (1154)	Top	TGCTGTAAGCATGGGATCATAGGTGGGTTTTGGCCACTGACTGACCCACCTATTCCCATGCTTA
		Bottom	CCTGTAAGCATGGGAATAGGTGGGTCAGTCAGTGGCCAAAACCCACCTATGATCCCATGCTTAC
Fbxw8	Inv-1 (432)	Top	TGCTGTCAGCAATCACCTTCCATGTCGTTTTGGCCACTGACTGACGACATGGAGTGATTGCTGA
		Bottom	CCTGTCAGCAATCACTCCATGTCGTCAGTCAGTGGCCAAAACGACATGGAAGGTGATTGCTGAC
	Inv-2 (556)	Top	TGCTGTCTTAATGTGTGCTCCTTGGCGTTTTGGCCACTGACTGACGCCAAGGAACACATTAAGA
		Bottom	CCTGTCTTAATGTGTTCCTTGGCGTCAGTCAGTGGCCAAAACGCCAAGGAGCACACATTAAGAC
Gnpda1	Inv-1 (13)	Top	TGCTGCTGGGAATAGTGTTCCAGGATGTTTTGGCCACTGACTGACATCCTGGAACTATTCCCAG
		Bottom	CCTGCTGGGAATAGTTCCAGGATGTCAGTCAGTGGCCAAAACATCCTGGAACACTATTCCCAGC
	Inv-2 (50)	Top	TGCTGTACGGTTCCTAATATACTTGGGTTTTGGCCACTGACTGACCCAAGTATTAGGAACCGTA
		Bottom	CCTGTACGGTTCCTAATACTTGGGTCAGTCAGTGGCCAAAACCCAAGTATATTAGGAACCGTAC
Lrguk	OBS	Top	TGCTGTTTAGATGAGATGTCAACAGCGTTTTGGCCACTGACTGACGCTGTTGATCTCATCTAAA
		Bottom	CCTGTTTAGATGAGATCAACAGCGTCAGTCAGTGGCCAAAACGCTGTTGACATCTCATCTAAAC
Map3k1	OBS	Top	TGCTGATATCCTCCGAGAGAGCTTGCGTTTTGGCCACTGACTGACGCAAGCTCTCGGAGGATAT
		Bottom	CCTGATATCCTCCGAGAGCTTGCGTCAGTCAGTGGCCAAAACGCAAGCTCTCTCGGAGGATATC
	Inv-2 (1214)	Top	TGCTGTTGACATGCGTGACACAAACTGTTTTGGCCACTGACTGACAGTTTGTGACGCATGTCAA
		Bottom	CCTGTTGACATGCGTCACAAACTGTCAGTCAGTGGCCAAAACAGTTTGTGTCACGCATGTCAAC
	Inv-1 (2037)	Top	TGCTGTTGACAAGGATAGTGTCTACAGTTTTGGCCACTGACTGACTGTAGACAATCCTTGTCAA
		Bottom	CCTGTTGACAAGGATTGTCTACAGTCAGTCAGTGGCCAAAACTGTAGACACTATCCTTGTCAAC
Nap1l4	Inv-1 (120)	Top	TGCTGTTGTCAAGACGTTCCTGCAAAGTTTTGGCCACTGACTGACTTTGCAGGCGTCTTGACAA
		Bottom	CCTGTTGTCAAGACGCCTGCAAAGTCAGTCAGTGGCCAAAACTTTGCAGGAACGTCTTGACAAC
	Inv-2 (299)	Top	TGCTGTTCTCTTGTCAAATAGAGGCTGTTTTGGCCACTGACTGACAGCCTCTATGACAAGAGAA
		Bottom	CCTGTTCTCTTGTCATAGAGGCTGTCAGTCAGTGGCCAAAACAGCCTCTATTTGACAAGAGAAC
Pdcd4	Inv-1 (235)	Top	TGCTGAACCGCTTCACTTCCATTGTCGTTTTGGCCACTGACTGACGACAATGGGTGAAGCGGTT
		Bottom	CCTGAACCGCTTCACCCATTGTCGTCAGTCAGTGGCCAAAACGACAATGGAAGTGAAGCGGTTC
	Inv-2 (439)	Top	TGCTGTTCATAAACACAGTTCTCCTGGTTTTGGCCACTGACTGACCAGGAGAAGTGTTTATGAA
		Bottom	CCTGTTCATAAACACTTCTCCTGGTCAGTCAGTGGCCAAAACCAGGAGAACTGTGTTTATGAAC
Pik3c2a	OBS	Top	TGCTGATAGAGTTAAAGAAACTTTGCGTTTTGGCCACTGACTGACGCAAAGTTTTTAACTCTAT
		Bottom	CCTGATAGAGTTAAAAACTTTGCGTCAGTCAGTGGCCAAAACGCAAAGTTTCTTTAACTCTATC
	Inv-1 (2525)	Top	TGCTGAAATAATGTCAAACGCAGGAGGTTTTGGCCACTGACTGACCTCCTGCGTGACATTATTT
		Bottom	CCTGAAATAATGTCACGCAGGAGGTCAGTCAGTGGCCAAAACCTCCTGCGTTTGACATTATTTC
	Inv-2 (2884)	Top	TGCTGATCACTAATGGCCTCCATCCAGTTTTGGCCACTGACTGACTGGATGGACCATTAGTGAT
		Bottom	CCTGATCACTAATGGTCCATCCAGTCAGTCAGTGGCCAAAACTGGATGGAGGCCATTAGTGATC
Pip5k1b	OBS	Top	TGCTGATTGTTCATCACCACAATACGGTTTTGGCCACTGACTGACCGTATTGTTGATGAACAAT
		Bottom	CCTGATTGTTCATCAACAATACGGTCAGTCAGTGGCCAAAACCGTATTGTGGTGATGAACAATC
	Inv-1 (581)	Top	TGCTGTCAAATGCATCCTCATGGCACGTTTTGGCCACTGACTGACGTGCCATGGATGCATTTGA
		Bottom	CCTGTCAAATGCATCCATGGCACGTCAGTCAGTGGCCAAAACGTGCCATGAGGATGCATTTGAC
	Inv-2 (896)	Top	TGCTGTGGAATAGAGGACTTTCTGCAGTTTTGGCCACTGACTGACTGCAGAAACCTCTATTCCA
		Bottom	CCTGTGGAATAGAGGTTTCTGCAGTCAGTCAGTGGCCAAAACTGCAGAAAGTCCTCTATTCCAC
Ppt2	Inv-1 (131)	Top	TGCTGTGTATGAACTGTCAAAGAGCCGTTTTGGCCACTGACTGACGGCTCTTTCAGTTCATACA
		Bottom	CTGTGTATGAACTGAAAGAGCCGTCAGTCAGTGGCCAAAACGGCTCTTTGACAGTTCATACAC
	Inv-2 (198)	Top	TGCTGAGATCAAGCACTGTCACCACAGTTTTGGCCACTGACTGACTGTGGTGAGTGCTTGATCT
		Bottom	CCTGAGATCAAGCACTCACCACAGTCAGTCAGTGGCCAAAACTGTGGTGACAGTGCTTGATCTC
Rab2b	Inv-1 (134)	Top	TGCTGCATCGATGTTGACCATACGTGGTTTTGGCCACTGACTGACCACGTATGCAACATCGATG
		Bottom	CCTGCATCGATGTTGCATACGTGGTCAGTCAGTGGCCAAAACCACGTATGGTCAACATCGATGC
	Inv-2 (110)	Top	TGCTGCAAACTCCACACCTATTGTGAGTTTTGGCCACTGACTGACTCACAATATGTGGAGTTTG
		Bottom	CCTGCAAACTCCACATATTGTGAGTCAGTCAGTGGCCAAAACTCACAATAGGTGTGGAGTTTGC
Tcf20	Inv-1 (1726)	Top	TGCTGTGAAGCGCCACACTTATAGGTGTTTTGGCCACTGACTGACACCTATAAGTGGCGCTTCA
		Bottom	CCTGTGAAGCGCCACTTATAGGTGTCAGTCAGTGGCCAAAACACCTATAAGTGTGGCGCTTCAC
	Inv-2 (18)	Top	TGCTGTTTCCGTGGTAACTGCTTTGCGTTTTGGCCACTGACTGACGCAAAGCATACCACGGAAA
		Bottom	CCTGTTTCCGTGGTATGCTTTGCGTCAGTCAGTGGCCAAAACGCAAAGCAGTTACCACGGAAAC
Tmem179b	Inv-1 (104)	Top	TGCTGACAAGGGACAGTTACCACCAAGTTTTGGCCACTGACTGACTTGGTGGTCTGTCCCTTGT
		Bottom	CCTGACAAGGGACAGACCACCAAGTCAGTCAGTGGCCAAAACTTGGTGGTAACTGTCCCTTGTC
	Inv-2 (207)	Top	TGCTGTAGAGTGCCAAGATGCCTGAAGTTTTGGCCACTGACTGACTTCAGGCATTGGCACTCTA
		Bottom	CCTGTAGAGTGCCAATGCCTGAAGTCAGTCAGTGGCCAAAACTTCAGGCATCTTGGCACTCTAC
